# A Multipurpose Microhaplotype Panel for Genetic Analysis of California Chinook Salmon

**DOI:** 10.1111/eva.70110

**Published:** 2025-05-13

**Authors:** Eric C. Anderson, Anthony J. Clemento, Matthew A. Campbell, Devon E. Pearse, Anne K. Beulke, Cassie Columbus, Ellen Campbell, Neil F. Thompson, John Carlos Garza

**Affiliations:** ^1^ Southwest Fisheries Science Center National Marine Fisheries Service, NOAA Santa Cruz California USA; ^2^ Institute for Marine Sciences University of California Santa Cruz California USA; ^3^ Department of Ocean Sciences University of California Santa Cruz California USA

**Keywords:** amplicon sequencing, genetic stock identfication, parentage‐based tagging, population assignment

## Abstract

Genetic methods have become an essential component of ecological investigation and conservation planning for fish and wildlife. Among these methods is the use of genetic marker data to identify individuals to populations, or stocks, of origin. More recently, methods that involve genetic pedigree reconstruction to identify relationships between individuals within populations have also become common. We present here a novel set of multiallelic microhaplotype genetic markers for Chinook salmon, which provide excellent resolution for population discrimination and relationship identification from a rapidly and economically assayed panel of markers. We show how this set of genetic markers assayed by sequencing 204 amplicons, in tandem with a reference dataset of 1636 individual samples from 17 populations, provides definitive power to identify all known lineages of Chinook salmon in California. The inclusion of genetic loci that have known associations with phenotype and that were identified as outliers in examination of whole‐genome sequence data allows resolution of stocks that are not highly genetically differentiated but are phenotypically distinct and managed as such. This same set of multiallelic genetic markers has ample variation to accurately identify parent‐offspring and full‐sibling pairs in all California populations, including the genetically depauperate winter‐run lineage. Validation of this marker panel in coastal salmon populations not previously studied with modern genetic methods also reveals novel biological insights, including the presence of a single copy of a haplotype for a phenotype that has not been documented in that part of the species range, and a clear signal of mixed ancestry for a salmon population that is on the geographic margins of the primary evolutionary lineages present in California.

## 1 Introduction

Genetic markers have long been used in the management of Pacific salmon, and salmon conservation and management have been at the forefront of a number of advances in molecular ecology, from data generation techniques (Clemento et al. 2011; Campbell et al. 2015; McKinney et al. 2017; Baetscher et al. 2018) to statistical methodology (Smouse et al. 1990; Anderson and Thompson 2002; Pella and Masuda 2006; Anderson 2010). Two broadly applicable techniques that have been actively fostered by the Pacific salmon research community are genetic stock identification (GSI: Milner et al. 1982; Beacham et al. 2004; Seeb et al. 2007) and parentage‐based tagging (PBT: Anderson and Garza 2006; Garza and Anderson 2007; Abadía‐Cardoso et al. 2013; Steele et al. 2013).

In the 1980s, electrophoretically detectable genetic variation, in the form of allozymes (Ayala and Powell 1972; Allendorf and Phelps 1981), was used to establish a program of GSI for Chinook salmon, *Oncorhynchus tshawytscha* (Milner et al. 1982). Extensive sampling revealed that these allozyme markers exhibited different allele frequencies among major lineages, or stocks, of Chinook salmon on the West Coast of the USA. These allele frequency differences make GSI possible, and Milner et al. (1985) soon showed that proportions of stocks in the Washington state coastal troll fishery could be estimated by GSI. Since that time, with the development of novel molecular markers, and now with increasing capacity to sequence genomic material, the scope and scale of GSI have expanded considerably.

A critical ingredient for GSI is the *reference dataset*, which consists of individuals sampled from across a range of populations and with each individual genotyped using the same panel of genetic markers. When individuals of unknown origin are genotyped at the same panel of genetic markers, the availability of the reference dataset makes it possible to do inference regarding the population of origin of the unknown individuals. Such reference datasets have seen widespread use and development in salmon management, where they are often called “genetic baselines,” and defined as, “databases of genotypes from breeding populations” (Seeb et al. 2007). Throughout, we will use “reference” or “reference dataset” and “baseline” interchangeably; however, a key point to keep in mind is that as genetic technologies evolve over time, reference datasets continue to be developed and refined to exploit the new technologies.

By using greater numbers of more variable markers than the allozymes available in the 1980s, it is possible to accurately identify the population of origin of individual fish, rather than simply estimating aggregated stock proportions. It is also possible to resolve populations of fish that are much more closely related than before. Furthermore, reference datasets with genotypes from hundreds of populations throughout the range of multiple species of salmon and other anadromous species (Seeb et al. 2007; Gilbey et al. 2018; Barclay and Habicht 2019) now exist and are routinely used to assign fish caught thousands of kilometers from their natal streams to their stock of origin. Applications include estimating fishery composition (Satterthwaite et al. 2015), providing real‐time information for genetics‐informed fishery closures (Beacham et al. 2004), assessing the spatial distribution of different stocks in the ocean (Urawa et al. 2009) and their temporal distribution in upstream migrations (Hess et al. 2014), and monitoring bycatch (Hasselman et al. 2016) or illegal captures (Wilmot et al. 1999) in marine fisheries.

Although sequencing costs continue to decline, they are high enough that there remains a tradeoff between reference baselines that include information from a large number of populations across a broad scale and those that have been tailored to distinguish between closely related populations on a smaller, regional scale. Because of cost considerations, reference baselines that include populations across a broad spatial scale may include only a few populations from each subregion. Furthermore, baselines tailored to a specific region often assemble markers that show allele frequency differences between the closely related—and hence difficult to resolve—stocks within the region. Consequently, regionally targeted baselines typically outperform broad‐scale baselines in resolving populations within the region.

Over the last decade, an additional genetic method, parentage‐based tagging, or PBT (Anderson and Garza 2005; Steele et al. 2019), has become established as an extremely valuable management tool for Pacific salmon. The availability of such family‐based methods adds another factor to consider when developing a GSI baseline. Since the first proposal (Anderson and Garza 2005) to replace or augment the coded‐wire tag program (Nandor et al. 2010) with PBT, it has been noted that one of the major advantages of a genetic program for PBT is that the genetic markers used for PBT could also be useful for GSI (and vice versa). Thus, any panel of markers to be used for GSI (or PBT) should also be evaluated on its utility for PBT (or GSI).

PBT has been remarkably successful in fisheries management, having been used for over a decade in the management of Chinook salmon and steelhead trout in major basins of the Columbia River (Steele et al. 2019; Horn et al. 2023), and having been employed to dramatically further our understanding of the genetic inheritance of key traits in salmonids (Abadía‐Cardoso et al. 2013; Beulke et al. 2023). However, PBT is just one subset of a whole family of statistical genetic methods employing relationship inference to learn about populations. For example, inference of the full siblings among a sample of fish provides information about the effective number of adults producing offspring (Waples and Waples 2011; Wang 2023), which can be a valuable source of information when sampling of the adults is not possible. Accordingly, marker panels should also be evaluated on their capacity to resolve full‐sibling relationships.

Chinook salmon are the largest of the Pacific salmonids and have historically been the target of extremely high‐value and culturally important fisheries (Myers et al. 1998). Moreover, because of their high degree of ecotypic variation, they have provided fishery opportunities in many different seasons and geographic locations (Healey 1991). However, recent declines in population sizes, from the Yukon River in the Arctic north to the southern extent of their range in California, have led to multiple fishery closures to protect less productive stocks (Lindley et al. 2009). The co‐occurrence of fish from relatively productive and relict populations reaches its paragon in California, with the largest remaining ocean fisheries for Chinook salmon targeting the Central Valley fall‐run stock that spawns in different tributaries of the same river basin as the highly endangered and phenotypically distinct winter‐run stock and the threatened spring‐run stocks (Satterthwaite et al. 2015).

Here, we present a reference baseline for Chinook salmon, focusing on the regional scale of rivers within the state of California, and particularly targeted to the complex population structure of Chinook salmon within the California Central Valley (CCV). Chinook salmon of the two main CCV river basins—the Sacramento and the San Joaquin—exhibit the greatest run‐timing diversity within the species. With four recognized ecotypes, delineated primarily on the basis of run timing (fall‐, late‐fall‐, winter‐, and spring‐run), adult Chinook salmon can be found migrating or residing in freshwater every month of the year in California (Fisher 1994).

In spite of their distinct phenotypes and life history patterns, the four ecotypes of Chinook salmon in the Central Valley are closely related (Clemento et al. 2014) and share recent common ancestry that is independent of ongoing migration between these lineages. As such, GSI has been particularly challenging in the CCV, with at least one of the distinct ecotypes, the late‐fall‐run, unresolvable with previous GSI marker sets and with unsatisfactory power for discriminating the protected spring‐run stocks from the harvested fall‐run stock (Seeb et al. 2007; Clemento et al. 2014).

We present a new reference baseline that includes 1636 Chinook salmon individuals from 17 collections that were genotyped at 204 loci distributed throughout the genome. This baseline is highly effective for GSI within California and provides ample power for PBT inference of other close relatives, thus enabling a highly effective and efficient integrated GSI/PBT monitoring and evaluation program (Garza and Anderson 2007; Beacham et al. 2021). We describe how we developed and compiled the markers, including several new markers identified through reanalysis of the whole‐genome sequencing data of Thompson et al. (2020), that are particularly effective at distinguishing between closely related groups of fish, such as late‐fall and fall‐run Chinook salmon in the CCV. We also provide a comprehensive analysis of this set of markers for inference of parents and siblings within different groups of populations.

## 2 Methods

### 2.1 Population Sampling

**FIGURE 1:**
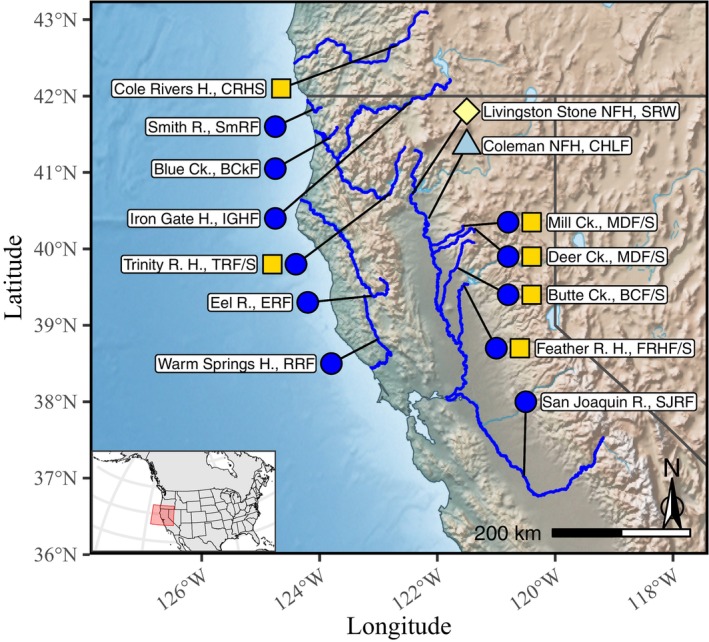
A map of sampling locations represented in the reference baseline. The colored shapes aside each location name represent the different run‐timing groups of Chinook salmon included in the collections from the location. Color and shape codes are as follows: 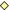 = Winter run, 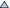 = Late‐fall run, 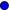 = Fall run, 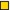 = Spring run. Names of each location are followed by the location codes (Table 1). Abbreviations used in location names as follows: Ck. = Creek; H. = Hatchery; NFH = National Fish Hatchery; R. = River.

Samples of fish for the reference baseline were compiled from 13 locations within California and one within Oregon. This set of populations and stocks includes all of the previously described lineages of Chinook salmon in the southern portion of the range and most of the populations that are known to have significant genetic differentiation in this region. These locations included sites in the two main tributaries (the Sacramento River and the San Joaquin River) within California's Central Valley, the two main tributaries (Klamath and Trinity Rivers) in the Klamath basin in Northern California, and three rivers of the California coast (Russian, Eel, and Smith). Of the 1636 total fish samples from 17 different populations, 727 fish from 10 collections were of the fall‐run ecotype, 498 fish from five collections were spring run, and 111 and 300 fish were of the winter‐run and late‐fall‐run ecotypes, respectively, each represented by a single collection (Figure 1, Table 1).

**TABLE 1:** Collections of fish in the reference baseline. The sampling months column shows a pictorial representation of the proportion of each collection sampled in each month. BCkF were sampled as juveniles; all other collections were of adults. Colors in this column correspond to run‐timing groups as described in the caption of Figure 1.

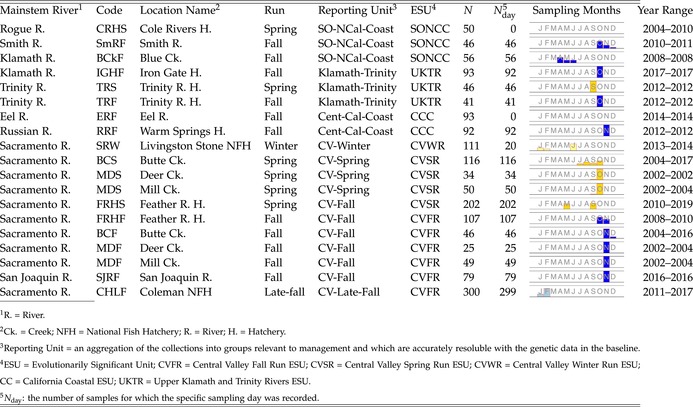

Abbreviations: CC = California Coastal ESU; Ck. = creek; CVFR = Central Valley Fall Run ESU; CVSR = Central Valley Spring Run ESU; CVWR = Central Valley Winter Run ESU; ESU = Evolutionarily Significant Unit; H. = hatchery; NFH = National Fish Hatchery; R. = river; UKTR = Upper Klamath and Trinity Rivers ESU.

^1^ Reporting Unit = an aggregation of the collections into groups relevant to management and which are accurately resolvable with the genetic data in the baseline.

^2^ $N_{\mathrm{day}}$: the number of samples for which the specific sampling day was recorded.

Collections in each location were separated into fish from the different adult migration‐timing ecotypes according to a variety of criteria. Winter‐run Chinook salmon are propagated at the Livingston Stone National Fish Hatchery for supplementation (released as juveniles into the river) and as a captive broodstock; sampling of this population was performed by hatchery staff during spawning. In Central Valley rivers with both spring‐run and fall‐run ecotypes (i.e., Mill, Deer, and Butte Creeks), previous research has documented different (but overlapping) ranges of spawning time (Julian date) for each ecotype (Fry 1961; Yoshiyama et al. 1998). As such, sampling targeted carcasses found in each of these rivers at times representative of their run‐timing designation, which were further confirmed using their genotypes within the region of strongest association (RoSA) on chromosome 28 (Thompson et al. 2020). In the San Joaquin River, in 2016, at the time of sampling, only fall‐run Chinook salmon adults were found; therefore, the samples could be categorized. Run‐timing designation at the Feather River Hatchery is more complicated, but it is managed by the hatchery practices in place: early returning fish in May and April (expressing the spring‐run life history) are visibly tagged and returned to the river. Subsequently, in the fall, when hatchery raceways are reopened, only fish with the visible tag are included as spring‐run broodstock, and the rest are included in the fall‐run broodstock. Putative spring‐run and fall‐run fish from these two broodstocks were sampled. Similar to broodstock collections in the CCV, samples from spring‐run and fall‐run fish in the Trinity River were selected on the basis of spawn timing at the Trinity River Hatchery, with migration‐timing ecotype confirmed by RoSA genotype. Elsewhere, adult samples were taken from live broodstock at Rowdy Creek (Smith River), Iron Gate (Klamath River) and Warm Springs (Russian River) Hatcheries, and juveniles were sampled from Blue Creek (Klamath River); all locations where only fall‐run fish have been documented. Spring‐run adult fish were sampled from the spring‐run broodstock at Cole Rivers Hatchery on the Rogue River. Samples from the Eel River were taken from live fish ascending a ladder in the upper basin and were confirmed as fall‐run ecotype by RoSA genotype.

In total, the samples in the baseline represent fish from five (Table 1) different Evolutionarily Significant Units (ESUs) of Chinook salmon (Waples 1991).

### 2.2 Genetic Markers

The genetic markers in the baseline include newly discovered microhaplotypes, SNP assays that were translated into amplicons, markers associated with phenotypes, and species‐ and sex‐specific markers. These genetic markers were compiled from several different efforts, as detailed in the sections below.

#### 2.2.1 Novel Microhaplotype Discovery

Candidate loci with multiple single‐nucleotide polymorphisms (SNPs) among multiple populations were identified using reduced‐representation genome sequencing of 2–4 Chinook salmon, each, from a number of populations ranging from California to British Columbia and the interior Columbia River (Table S1). Genomic DNA was extracted using Qiagen DNeasy 96 Blood and Tissue Kits on a BioRobot 3000 (Qiagen Inc). Double‐digest restriction‐site associated DNA sequencing (ddRAD) with different size selections (400 and 500 bp fragments) was used to produce a broad array of locus targets. Library preparation and sequencing methods followed those of Peterson et al. (2012) with the modifications described in Baetscher et al. (2018). Sequencing was performed on a MiSeq (Illumina Inc) using 2 × 300‐cycle paired‐end sequencing. Stacks v1.45 (Catchen et al. 2013) was used to analyze the ddRAD sequence data. As there was not a well‐assembled Chinook salmon genome at the time, the ustacks module of Stacks was used to assemble reads de novo into RAD loci. Initial filtering retained RAD loci with two or more SNPs present in at least 10 samples. After this initial filtering, there were 3931 RAD loci retained in the ddRAD data with 400‐bp size selection and 4794 retained in the data with 500‐bp size selection

The 8725 discovered RAD loci were filtered down to a set of markers suitable for conversion to amplicon‐sequencing assays. Gene regions were selected from the RAD loci that (1) contained multiple SNPs within a 100‐bp window and (2) showed haplotype variation among the winter run and at least one other population. A 100‐bp window was used because the success of multiplex PCR and MiSeq sequencing is diminished in amplicons larger than 100 bp. Primer3 software (Untergasser et al. 2012) was used to design primers for 229 gene regions that met these criteria. These 229 gene regions were tested for PCR‐amplification and consistency in a separate sample of 288 Chinook salmon: 96 SRW fish and 48 each from the Entiat River (a Columbia River tributary), TRH, FRHS, and CRLF. Eight GT‐seq amplifications were made and these were sequenced on eight runs of a MiSeq sequencer using a 2 × 75‐cycle kit to identify problematic loci and to optimize primer dilutions. The sequences from each run at each locus were mapped to the de novo reference sequences (produced by Stacks) with bwa mem (Li and Durbin 2009). Subsequently, alignments were sorted with SAMtools (Li et al. 2009) and provided to FreeBayes (Garrison and Marth 2012) for variant calling. FreeBayes output was filtered to remove indels and retain variants with a Phred‐scaled quality score of 30 or greater and a read depth of 10 or higher. Further filtering and analysis of the loci, including assessment of Hardy–Weinberg equilibrium (Hardy 1908) was conducted in MICROHAPLOT (Ng et al., https://doi.org/10.5281/zenodo.820110). Loci that produced more than two haplotypes within an individual, and those clearly violating Hardy–Weinberg equilibrium within populations were removed.

#### 2.2.2 Conversion of Existing SNPtype Assays

A number of SNP markers from an earlier discovery effort (Clemento et al. 2011) had already been validated as useful for GSI and PBT in California (Clemento et al. 2014). These markers were originally assayed using TaqMan (Life Technologies Corp.) probes or SNPtype assays (Standard BioTools Inc.). These markers were typed using amplicon sequencing, following the methods in Campbell et al. (2015) with the addition of the Illumina small RNA sequencing primer and read 2 sequencing primer to the SNPtype Specific Target Amplification Primer and Locus Specific Primers respectively. Loci were removed that were frequently inconsistent with SNPtype assays, had poor amplification, or had excessive amplification and were subsequently overrepresented in the sequencing library. FreeBayes was used to identify all SNPs (additional to the TaqMan‐assay targets) along the fragment, which facilitated scoring fragment microhaplotypes when multiple SNPs were present along the read (Baetscher et al. 2018). The resulting genetic markers are hereafter referred to as “SNPlicons” to record their origin as TaqMan assays in a previous baseline

#### 2.2.3 Identifying the Late‐Fall Associated Region (LFAR) and Designing Markers Therein

Loci with large allele frequency differences between late‐fall and fall‐run Chinook salmon were identified with a simple case–control genome‐wide association study (GWAS) using the low‐coverage whole‐genome sequencing data of Thompson et al. (2020), mapped to the Otsh_v1.0 Chinook salmon assembly (GenBank accession GCA_002872995.1). We conducted two separate association‐study comparisons. The first involved the 16 late‐fall‐run fish as cases with 16 fall‐run fish from the Feather River Hatchery as controls. The second involved the same 16 late‐fall fish as cases, but used 16 fall‐run fish from the San Joaquin River as controls. The association studies were performed for each chromosome with ANGSD (Kim et al. 2011; Korneliussen et al. 2014), using the following command: angsd ‐yBin {ybin} ‐r {chrom} ‐minMapQ 30 ‐minQ 20 ‐minInd 12 ‐doAsso 1 ‐GL 1 ‐doMajorMinor 1 ‐doMaf 1 ‐SNP_pval 1e‐6 ‐out {prefix} ‐bam {bamlist}, where {ybin} is the path to a file indicating the cases and controls, {chrom} indicates the chromosome name, {prefix} is the prefix to use for output files, and {bamlist} is the path to the text file holding paths to the binary alignment map (BAM) files of aligned sequence data, one for each individual, ordered as in {ybin}

The GWAS *p* values were compared relative to the commonly used significance threshold for GWAS of 5 × 10^−8^ (Chen et al. 2021). Only a single site exceeded that threshold in both comparisons and it occurred atop a peak in association scores on chromosome 34 (see Section 3). Candidates for follow‐up genotyping were identified in the peak by including the 10 SNPs in that peak with the lowest association *p* values, and supplementing those with additional SNPs that showed:

- An estimated absolute allele frequency difference between late‐fall and all 32 fall‐run fish, |*d*| > 0.75, with an approximate lower confidence interval of |*d*| > 0.5, or
- |*d*| > 0.5 with a lower confidence interval > 0.25 and with either fall‐run or late‐fall run being nearly fixed for one of the alleles, or
- An annotation from snpEff (Cingolani et al. 2012) of “High” or “Moderate,” or
- |*d*| > 0.48 and a lower confidence interval of > 0.25 and a genomic coordinate > 2.2 Mb.

The final condition was implemented in order to gather several SNPs with large allele frequency differences that were adjacent to, but not directly within, the main peak of association, so as to possibly learn about recombination in the region.

We designed amplification primers for the candidates using Primer3 (Koressaar and Remm 2007; Untergasser et al. 2012) and tested amplification in a subset of Chinook salmon samples. The primer pairs that amplified sequences that reliably mapped back to the association‐peak region were typed in a separate collection of 1638 Sacramento River Chinook salmon sampled from the fish trap below Keswick Dam, including 1169 winter run, 281 spring run, and 188 fall/late‐fall run. We divided the fish identified from the 125‐locus GSI dataset of Thompson et al. (2020) as fall/late‐fall into fall run and late‐fall run according to their sampling date at Keswick: 99 fish arriving before December 26 were classified as fall run and 89 fish arriving after December 26 were classified as late‐fall run. The allele frequencies of the target SNPs at the remaining amplicons were estimated for each of these four groups: winter, spring, fall, and late‐fall, and amplicons showing large allele frequency differences between late‐fall fish and all other runs were retained.

#### 2.2.4 Winter‐Run Associated Polymorphisms (WRAPs)

Using the whole‐genome sequencing data of Thompson et al. (2020), we also sought genomic regions that might provide diagnostic markers for the Sacramento winter‐run Chinook salmon. This ecotype is highly differentiated from all other Chinook salmon populations throughout its genome, which complicates the process of finding diverged genome regions using a simple association study, with only 16 samples, as done to identify the LFAR. Consequently, we pursued a bespoke analysis to find regions expected to have large allele frequency differences between the winter‐run and all other ecotypes of Chinook salmon in California's Central Valley. This analysis screened for regions with large allele frequency differences across large blocks of the genome and is described in Appendix S1: Section S1.

#### 2.2.5 Adult‐Migration‐Timing Associated Markers

We include six amplicons that capture the eight SNPs within the “region of strongest assocation” (RoSA) between spring‐run and fall‐run fish, near the ROCK1 and GREB1L genes, on Chromosome 28 listed in table S3 of Thompson et al. (2020). We add three more amplicons in the same RoSA region to the panel, as well as one more amplicon that allows genotyping of snp670329, which was discovered and described by Thompson et al. (2019). Including all these markers in the panel allows them to be routinely typed, creating a database for understanding the spatial and temporal distribution of the alleles in this genomic region; however, these markers are omitted from likelihood calculations for GSI, since their distribution across populations in the Feather River has been clearly and evidently influenced by hatchery practices (see Section 3), it is not necessary to include them for accurate assignment, and because the 12 SNPs within these three amplicons are typically in profound linkage disequilibrium (LD) making them inappropriate for use as separate markers in most software for population assignment or GSI, that typically assumes markers are not in LD within the component populations of the baseline.

#### 2.2.6 Additional Phenotypically Associated Markers

The baseline also includes three amplicons with genetic variation in two genes, VGLL3 and SIX6, found to be associated with phenotypes related to reproductive maturity in both Atlantic (Barson et al. 2015) and Pacific (Waters et al. 2021) salmonids. They provide additional variation for population genetic analyses and to monitor populations for potential phenotypic patterns.

We include an assay from a novel locus that targets the sex‐determining region (*sdY*) on the Y chromosome in Chinook salmon. We designed this assay to target the SNP described by Bertho et al. (2022) that differentiates the functional *sdY* and a nonfunctional *sdY* pseudogene. Examination of whole‐genome sequence data in multiple populations of California Chinook salmon (Thompson et al. 2020) shows that this marker characterizes genetic sex in diverse California salmon populations more accurately than previously published assays (Von Bargen et al. 2015). Finally, marker Ots_coho001_05_32691399, which is fixed for alternate alleles in coho salmon and Chinook salmon, is included to diagnose when samplers have unwittingly collected coho salmon.

### 2.3 Localizing Markers Within the Genome

The novel microhaplotype markers and the SNPlicon targets were developed prior to the advent of a well‐assembled genome for Chinook salmon. As a consequence, many of these markers had been used previously without certainty about their location in the genome. After designing and validating an amplicon‐sequencing approach to type both of these groups of markers, we identified their locations within the genome by mapping the consensus sequences (roughly 100–300 bp) around the markers to both the Chinook salmon version 1 (Otsh_v1.0, GenBank accession GCA_002872995.1) and version 2 (Otsh_v2.0, GenBank accession GCA_018296145.1) genome assemblies, using both bwa mem and the BLAST‐like Alignment tool, BLAT (Kent 2002). A similar mapping exercise was performed for the remaining markers to report their locations in both Otsh_v1.0 and Otsh_v2.0.

### 2.4 Amplicon Sequencing, Microhaplotype Calling, and Further Quality Control

Amplicon sequencing was performed with the Genotyping‐in‐Thousands‐by‐sequencing (GT‐seq) method (Campbell et al. 2015). All loci were multiplexed with primer concentrations ranging from 0.083 to 0.5 μM to increase uniformity of read depths across all loci. Sample normalization was performed using the SequalPrep Normalization Plate Kit (Applied Biosystems) according to the manufacturer's instructions. Samples were pooled by plate and purified using Agencourt AMPure XP magnetic beads. Libraries were quantified either by qPCR with the NEBNext Library Quant Kit for Illumina (New England BioLabs) or with the Qubit dsDNA HS Assay Kit (Thermo Fisher Scientific) before dilution and pooling for sequencing. All samples were sequenced on a MiSeq using 2 × 75‐cycle paired‐end sequencing protocols. Primer sequences and concentrations for making the primer pool are in Supplemental Data S1.

For evaluation of candidate loci, dual‐barcoded sequences were used to identify individual tissue samples using the MiSeq analysis software (Illumina). The paired‐end sequence reads were joined together using Fast Length Adjustment of SHort reads (FLASH: Magoč and Salzberg 2011) and mapped to an indexed sequence reference created from the Stacks target loci (for the novel microhaplotypes, SNPlicons and RoSA markers) or from the entire Otsh_v1.0 genome (for the LFAR, WRAP, VGLL3, and Six6 markers) using bwa mem. Mapped reads were converted to BAM files using SAMtools, and FreeBayes was used to call variants.

For genotyping of baseline samples, final filtering at each locus required a read depth of 20 and a read depth ratio of 0.30 (i.e., for heterozygotes the lower read depth haplotype must have 30% of the total reads at that locus). MICROHAPLOT was used to call alleles and genotype processing was performed with R scripts.

Each locus was assessed for the presence of null alleles using the R package “whoa” (https://github.com/eriqande/whoa; see Hendricks et al. 2018).

### 2.5 Population Genetic Analyses

Basic data summaries were calculated for each population/collection in the reference baseline using simple operations in the R programming language, version 4.3 (R Core Team 2024). Summaries provided are:

- the total fraction of missing genotypes, M;
- the average number of alleles per locus in each collection with all collections downsampled to the smallest number of genotyped individuals at each locus, ${\bar{N}}_{A,\mathrm{ss}}$;
- the fraction of polymorphic loci in each collection after subsampling to the smallest number of genotyped fish, ${\bar{P}}_{\text{poly},\mathrm{ss}}$;
- the expected heterozygosity as the average over loci of one minus the frequencies of homozygotes at each locus expected from the estimated allele frequencies, ${\bar{H}}_{\exp}$; and
- the observed heterozygosity as the average over loci of the fraction of heterozygous genotypes, ${\bar{H}}_{\mathrm{obs}}$.

When subsampling each collection to the minimum number of genotyped fish across all collections at a locus, individuals were subsampled without replacement, taking the average of 1000 different random subsamples.

We calculated Weir and Cockerham (1984)'s pairwise $F_{\mathrm{ST}}$ between all collections, using the pp.fst function from the R package “hierfstat” (Goudet and Jombart 2022). Population structure in the data was evaluated using the program STRUCTURE (Pritchard et al. 2000; Falush et al. 2003). For this analysis, we omitted the run‐timing associated markers. We performed 20 replicate runs at each value of the assumed number of subpopulations, K, in $\left\{2,3,4,5,6,7\right\}$ using default settings of the program. STRUCTURE output was summarized and visualized using CLUMPAK (Kopelman et al. 2015).

### 2.6 Power for Genetic Stock Identification and Population Assignment

To assess the power of the marker panel for GSI, we used the R package “rubias” (Moran and Anderson 2019). The function self_assign() was used to assign each individual in the reference baseline to one of the collections within the baseline. Each fish was removed from the reference baseline when calculating the likelihood that it originated from the collection it actually belongs to, using a classical leave‐one‐out approach to eliminate the inflated estimates of power that might occur without such a procedure (Anderson et al. 2008). The scaled likelihoods of collection membership returned by self_assign() were summed within reporting units, and fish were assigned to the reporting unit with the largest value of that sum. Subsequently, fish were assigned to the collection within that reporting unit with the highest scaled likelihood. We considered two thresholds for assignment. In the first, all fish are assigned, in the second only fish with a summed (i.e., summed over all collections in a reporting unit) scaled likelihood greater than 0.8 were assigned.

Finally, we investigated the matrix of assignments and misassignments broken down according to the genotype of the fish at the markers within the RoSA. RoSA genotype (EE, EL, or LL, see Thompson et al. 2020) was assessed from the concordance of the genotypes at all eight markers described in Thompson et al. (2020), requiring that no genotypes be missing within an individual from any of those eight markers and discarding data from 12 individuals in which the E or L haplotypes had clearly recombined.

### 2.7 Power for Relationship Inference

To explore the utility of the marker panel for relationship inference, we used the R package “CKMRsim” (https://github.com/eriqande/CKMRsim) to estimate the false positive rates (FPRs) expected at different values of the false negative rate (FNR) for a variety of different pairwise relationships in different populations. For these purposes, collections were divided into 10 different groups. The members of each group are relatively, genetically similar, but the groups do not necessarily correspond to reporting units, because, in some cases, FPRs and FNRs are desired for a single collection. The assumed genotyping error rate was 1% per locus and the genotyping error model was the default “True‐genotype‐independent” model.

For errors of misidentifying unrelated (U) pairs as parent‐offspring (PO), full‐sibling (FS), or half‐sibling (HS) pairs, we used importance sampling to estimate the very small FPRs. The FNRs were estimated in each case using simple Monte Carlo taking account of the physical linkage of the markers by inputting their genomic positions into “CKMRsim” and using the package's interface to the MENDEL (Lange et al. 2013) software to simulate genotypes of related pairs in the presence of physical linkage. For errors of misidentifications between the relationship types of avuncular (AN: aunt‐niece, uncle‐nephew, etc.), PO, FS, and HS, we used regular Monte Carlo to estimate the FPRs and FNRs, because importance sampling cannot be used in those cases. Since there are expected to be far fewer such relationships than the number of unrelated pairs, the relevant FPRs are large enough that they can be reliably estimated (at least down to rates of 10^−3^) without importance sampling. For AN, FS, and HS, both the FPRs and FNRs were estimated while taking account of physical linkage of the markers, as described above.

## 3 Results

### 3.1 Genetic Markers

The number of markers and variants obtained from each of our discovery efforts appears in Table 2. Further information, including genomic location, consensus sequence, and primer sequences, of all the amplicons used in the reference dataset is available in Data Supplement S1. A graphical overview of genomic coordinates is found in Figure S1.

**TABLE 2:** Summary of amplicons in the reference dataset. Source denotes which discovery effort yielded the amplicon. *N* (amplicon) is the number of amplicons and *N* (variant) is the total number of variant sites assayed within the amplified sequence of all the amplicons. Source abbreviations are as follows: coho = distinguishes coho from Chinook salmon; lfar = late‐fall‐run associated polymorphisms; mhap = novel microhaplotype discovery; rosa = adult‐migration‐timing associated markers; scon = conversion of existing SNPtype assays (the “SNPlicons”); sexy = sexID marker; sixx and vgll = amplicons targeting the SIX6 and VGLL3 genes, respectively; wrap = winter‐run associated polymorphisms.

Type	*N* (amplicon)	*N* (variant)
mhap	106	458
scon	78	200
rosa*	10	22
wrap	3	7
lfar	2	6
sixx	2	2
coho*	1	2
sexy*	1	
vgll	1	1

*Note:* The * denotes markers routinely excluded from likelihood calculations for GSI.

#### 3.1.1 Additional Microhaplotype Discovery

Of 229 tested candidates from our novel microhaplotype discovery process, 125 markers were retained (Thompson et al. 2020) for use in stock identification within the Klamath Basin. However, further evaluation of those markers in other populations led to the removal of 12 loci with apparent null alleles in the winter‐run population, two that were monomorphic in the winter‐run, despite having multiple alleles in other populations, and an additional five loci that were difficult to score in the routine application of the baseline. This left 106 novel microhaplotype amplicons in the current panel.

#### 3.1.2 Conversion of Existing SNPtype Assays

Of the 96 existing SNPtype assays, we were able to convert 78 into reliable amplicon‐sequenced assays that were included in the panel.

#### 3.1.3 Late‐Fall Associated Region Markers

The case–control association identified only a single SNP with *p* < 5 × 10^−8^ in the comparison between late‐fall run and both the FRH fall‐run and the San Joaquin fall run. This SNP was within a single evident peak on chromosome 34 with large allele frequency differences between Central Valley late‐fall and fall‐run fish. This peak was evident both in the comparison of late‐fall fish to fall‐run fish in the Feather River Hatchery and of late‐fall fish to the fall‐run fish in the San Joaquin River (Figure S2)

The Manhattan plot of Figure S2 shows sporadic SNPs with low association *p* values. These are not shared between the two different late‐fall to fall comparisons and were concentrated in areas of poor mapping, suggesting that their *p* values were artifactual. By contrast, a large number of sites near the prominent peak on chromosome 34 had large allele frequency differences between late‐fall and fall run. Taking the 10 SNPs with the lowest association *p* values, and including additional ones from the filtering criteria given in Section 2, yielded 49 candidate markers for which we designed primers for amplification (Figure S3). Only 8 of these 49 primer pairs yielded reliable amplification and mapping. Of these 8, two of the SNPs showed inconsistent genotyping and were removed from consideration. Of the remaining 6, only 3 showed marked allele frequency differences (> 0.7) between late‐fall and fall run. These three SNPs (located at: Chr34:828,768, Chr34:865,057, and Chr34:1,063,084 in the Otsh_v1.0 assembly) also had pronounced differences in allele frequency between late‐fall and spring or winter run.

Analysis of the mapping of amplicons for Chr34:828,768 showed that many (≈89%) of the reads were off‐target (aligning to other chromosomes, etc.), making it costly (in terms of sequencing effort) to include the marker in the baseline panel. Consequently it was dropped from the panel. The two remaining loci, which are at coordinates Chr34:865,057, and Chr34:1,063,084 in the Otsh_v1.0 assembly are at coordinates Chr34:954,054 and Chr34:1,151,868 in the Otsh_v2.0 assembly, and the frequency of the different alleles at the two remaining loci within different reporting units in the baseline are shown in Table 3.

**TABLE 3:** Allele frequencies across reporting units of the two late‐fall associated markers. Frequencies are given for the alleles most common among late‐fall Chinook: Nucleotide G at locus Chr34:954,054 and A at Chr34:1,151,868. *N* is the total number of gene copies available for estimating the allele frequency. Genome coordinates of SNPs are from Otsh_v2.0.

Reporting unit	Chr34:954,054		Chr34:1,151,868	
	Freq.	*N*	Freq.	*N*
CV‐Late‐Fall	0.750	516	0.733	592
CV‐Fall	0.054	910	0.030	908
CV‐Spring	0.023	396	0.003	396
CV‐Winter	0.005	222	0.005	222
Cent‐Cal‐Coast	0.000	92	0.004	278
Klamath‐Trinity	0.000	172	0.000	358
SO‐NCal‐Coast	0.000	192	0.000	304

#### 3.1.4 Winter‐Run‐Associated Polymorphisms

Initial examination of sequence data in the 16 winter‐run fish and 84 non‐winter‐run fish revealed a number of SNPs that were potentially diagnostic, but none were fixed for alternate alleles in a larger sample. Nonetheless, three of these markers had large frequency differences between the groups and are included in the reference baseline. The process of discovering them is described in Appendix S1: Section S1.

### 3.2 Localizing Markers Within the Genome

Of the 184 loci that originated from our novel microhaplotype discovery or from SNPtype assays, 179 of them were mapped by both bwa mem and BLAT to exactly the same location on an assembled chromosome in the Otsh_v2.0 genome. Of the remaining five loci, one of them was mapped to the same location by BLAT and bwa mem, but the alignments differed in length; two of them had a single secondary alignment on a different chromosome that was identical in both bwa mem and BLAT; one of them had only a fragment mapping to the secondary alignment; and one of them had multiple nonprimary alignments (Appendix S1).

Mapping to Otsh_v1.0 was similar, except that a greater number of markers mapped to unplaced scaffolds in Otsh_v1.0 than in Otsh_v2.0, likely reflecting the more complete nature of the second assembly.

### 3.3 Population Genetic Summaries

The total number of alleles per locus varied between 1 and 10 (Figure S4), with the only locus bearing a single allele in Chinook salmon being the species‐specific marker Ots_coho001_05_32691399. Biallelic loci were most common, at 66, but two‐thirds of the microhaplotype markers showed more than two alleles, which has been shown to provide significantly more power for relationship inference than a comparably sized panel of biallelic SNPs (Baetscher et al. 2018).

The population‐genetic summary statistics are presented in Table 4.

**TABLE 4:** Simple population genetic summaries. All quantities are means over loci. M is the total fraction of missing genotypes; ${\bar{N}}_{A,\mathrm{ss}}$ is the average number of alleles and ${\bar{P}}_{\text{poly},\mathrm{ss}}$ the fraction of polymorphic loci, after subsampling to the smallest sample size per locus; ${\bar{H}}_{\exp}$ and ${\bar{H}}_{\mathrm{obs}}$ are expected and observed heterozygosity, respectively. Population codes are as given in Table 1.

Code	M	${\bar{N}}_{A,\mathrm{ss}}$	${\bar{P}}_{\text{poly},\mathrm{ss}}$	${\bar{H}}_{\exp}$	${\bar{H}}_{\mathrm{obs}}$
CRHS	0.008	2.60	0.95	0.398	0.399
SmRF	0.021	2.60	0.95	0.401	0.394
BCkF	0.041	2.59	0.96	0.393	0.382
IGHF	0.028	2.37	0.93	0.356	0.363
TRS	0.036	2.37	0.92	0.353	0.348
TRF	0.031	2.37	0.92	0.352	0.351
ERF	0.022	2.51	0.95	0.364	0.356
RRF	0.037	2.64	0.96	0.398	0.391
SRW	0.338	2.32	0.88	0.338	0.329
BCS	0.046	2.52	0.92	0.379	0.373
MDS	0.082	2.55	0.94	0.394	0.397
FRHS	0.108	2.61	0.95	0.397	0.392
FRHF	0.029	2.63	0.96	0.394	0.398
BCF	0.123	2.60	0.94	0.385	0.370
MDF	0.130	2.59	0.94	0.386	0.383
SJRF	0.012	2.57	0.94	0.384	0.380
CHLF	0.068	2.53	0.94	0.386	0.384

The total fraction of missing data by collection varied from a low of 0.008 in CRHS to a high of 0.338 in SRW, the latter due to a tranche of samples from degraded carcasses. The average number of alleles per locus, standardized to the smallest sample size, ranged from 2.32 (SRW) to 2.64 (RRF), with SRW also having the smallest value of the standardized fraction of polymorphic markers at 0.88 and RRF sharing the highest value (0.96) with FHRF and BCkF. SRW also had the lowest values for expected and observed heterozygosity: 0.338 and 0.329, respectively, while the highest for expected heterozygosity was 0.401 (SmRF) and for observed heterozygosity, 0.399 (CRHS).

**FIGURE 2:**
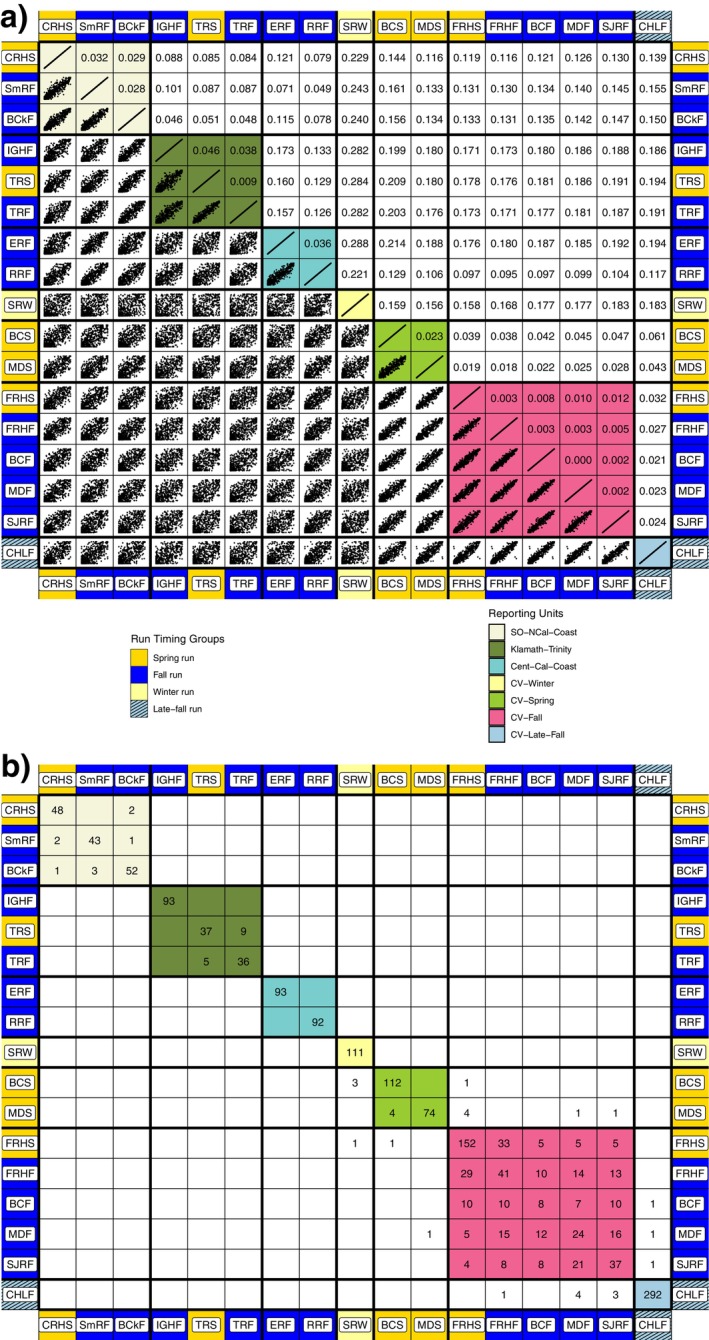
(a) Pairwise $F_{\mathrm{ST}}$ values and allele frequencies, and (b) self‐assignment rates. The codes of the populations appear on the outer margins of the table atop cells colored according to the run‐timing group of the collection. Interior cells are colored according to reporting unit. In (a), for $F_{\mathrm{ST}}$ values, the upper triangle was computed using all the genetic markers except for those in the RoSA on chromosome 28. Each cell in the lower triangle shows a small scatterplot of estimated allele frequencies in the collections—the x value is allele frequencies from the collection in the column and the y value is from the collection in the row. Frequencies for all alleles are shown, with values within each cell ranging from 0 to 1. These frequencies were estimated by, for each locus, randomly subsampling individuals from each collection to the minimum number of observed alleles in any collection at the locus. In (b), for self‐assignment tallies, the row corresponds to the true source collection and the column corresponds to the assigned collection. All individuals were assigned here, using a maximum‐scaled‐likelihood rule to reporting unit and to collection within reporting unit as described in the text. For the assignment matrix resulting when only fish with a scaled likelihood > 0.80 to reporting unit are assigned, see Figure S6.

The estimated pairwise $F_{\mathrm{ST}}$ values between the collections varied from a low of 0.0 to a high of 0.288 (Figure 2a). Notably, most of the largest values of $F_{\mathrm{ST}}$ occurred between SRW and another collection.

**FIGURE 3:**
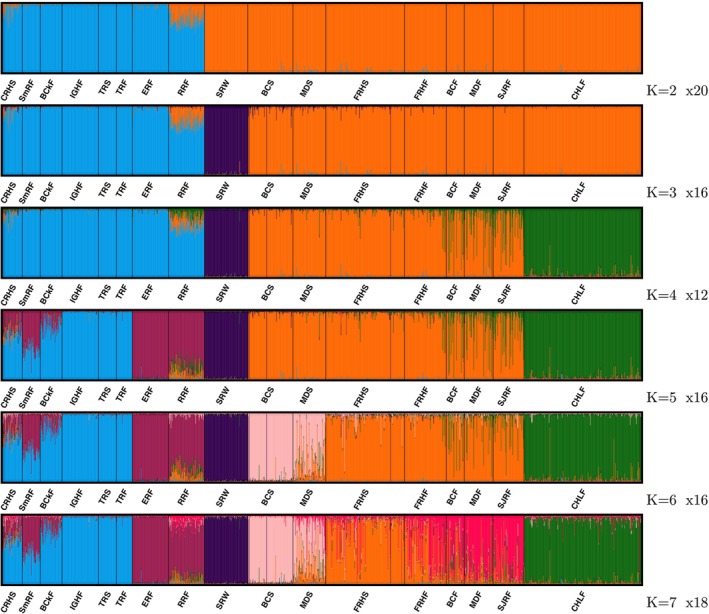
Major modes found by STRUCTURE and summarized by CLUMPAK out of 20 replicates for each value of *K*, the assumed number of subpopulations. *K* value is listed at lower right of each barplot, followed by the number of replicate runs of structure that converged to this major mode.

At values of *K* from 2 to 7, clusters identified by the program STRUCTURE generally corresponded to groupings of related populations, and confirmed a priori knowledge about which collections in the baseline could and should be grouped together into reporting units (Figure 3). At every value of *K*, CLUMPAK discerned at least 12 of 20 replicates in the major mode (Figure 3). Clustering solutions in the minor modes generally converged on a single alternative solution and appear in Figure S5.

The late‐fall‐run collection (CHLF) appears as a separate cluster at *K* values as low as 4, with the inclusion of the LFAR markers. This is notable, since accurately discriminating between the late‐fall and fall‐run Chinook salmon of the Central Valley has been impossible with previously used genetic marker sets. Another noteworthy feature appears at *K* = 7, with STRUCTURE separating the fall‐run reporting unit into two clusters that do not correspond exactly with the individual collections within the reference baseline. For example, fish from the cluster that predominates in the FRHS collection are also found in the FRHF and MDS collections.

### 3.4 Power for Genetic Stock Identification and Population Assignment

Leave‐one‐out cross‐validation (self‐assignment) analysis demonstrates that this reference baseline provides a high degree of accuracy for distinguishing the major groups of Chinook salmon in California. The results of the analysis are summarized in a self‐assignment matrix (Figure 2b). This figure shows the collections divided into the seven different reporting groups: SO‐NCal‐Coast, Klamath‐Trinity, Cent‐Cal‐Coast, CV‐Winter, CV‐Spring, CV‐Fall, and CV‐Late‐Fall. These groupings correspond largely to the divisions in the data found by STRUCTURE, and they also correspond with divisions among the stocks as defined for management, and, as seen in Figure 2b, they correspond with groups of populations that can be reliably distinguished from one another for population assignment.

Overall, of the 1636 fish in the reference baseline, 1612 (98.5%) were correctly assigned to their reporting unit of origin, while 24 (1.5%) were incorrectly assigned. Four of those 24 misassignments, which involved fish being incorrectly assigned to SRW, were clearly fish from SRW that had been incorrectly sampled into a different collection, likely due to straying (this is evident because SRW are so distinct that it is highly improbable they would be incorrectly identified). Of the remaining 20 misassignments, 8 involved fish from the CHLF (late‐fall run) collection being misassigned to the CV‐Fall reporting unit, and 6 were fish from the MDS (Mill‐Deer spring run) collection being assigned to the CV‐Fall reporting unit. It is possible that some of these misassignments represent CV‐Fall fish that were incorrectly sampled as CHLF or MDS; however, unlike with SRW, we cannot conclude that confidently, as there is some degree of overlap in likelihood values between the two.

While fish from CHLF were found to misassign at a rate of 2.7% (8/300) and MDS at a rate of 7.1% (6/84) to CV‐Fall, misassignment in the other direction occurs at a much lower rate—that is, fish from the CV‐Fall are misassigned to CHLF at a rate of only 0.6% (3/508) and to the CV‐Spring reporting unit (to which MDS belongs) at a rate of only 0.4% (2/508). When assignments are only accepted with a scaled likelihood greater than 0.8, 10 incorrect assignments and 10 correct assigments are discarded. Thus, at the 0.8 threshold, the total fraction of correct assignments is 99.1% (Figure S6). Notably with the > 0.8 criterion, the misassignment rates from the abundant CV‐Fall reporting unit to CV‐Late‐Fall or to CV‐Spring both drop below 0.2% (1/502).

As seen in previous studies, FRHS, the Feather River Hatchery spring‐run stock, is considerably differentiated from the remaining spring‐run stocks in the Central Valley (MDS and BCS). This is reflected in the low misassignment rates of FRHS fish to the CV‐Spring and is also evident in the STRUCTURE results. We note, however, that the RoSA markers provide the differential signal necessary to distinguish Feather River spring‐migrating fish from the other CCV stocks with a predominantly fall‐run genomic background.

FRHS fish are produced by selecting spring‐tagged fish that again return in fall, when the hatchery ladder reopens. This results in untagged spring run fish not encountered during the spring trapping season being used as broodstock for the fall‐run program. This is evident when the assignment matrix is enumerated in terms of RoSA genotypes (Figure S7). In this context, it is clear that the “fall‐run” hatchery program in the Feather River generates many spring‐run fish and RoSA heterozygotes. Similarly, in other regions, such as the Trinity River, the RoSA markers are the only reliable means to distinguish fall‐ and spring‐run fish, as they otherwise share genomic background.

In California, only the Sacramento and Klamath basins have documented spring‐run salmon, and their historical occurrence in other basins has been unclear; however, we identified three additional copies of a recombinant haplotype that carries half of the RoSA SNPs from both the E and L lineages in the Eel and Russian rivers. No other clear recombinants were identified in the study populations, suggesting that it arose in the California Coastal Chinook Salmon lineage and providing further evidence of the past presence of RoSA haplotypes associated with early migration.

### 3.5 Power for Relationship Inference

The “CKMRsim” power analyses for this set of markers demonstrates that they have ample variation for accurate identification of parent‐offspring (PO) and full‐sibling (FS) pairs in almost all realistic situations. The predicted FPRs from unrelated pairs for both the parent‐offspring (PO) and full‐sibling (FS) relationships are exceedingly low for all population groups, even when using stringent false negative thresholds as low as 0.05 (Figure 5a). For example, the FPR for FS identification is less than one error in 10 million comparisons of potential siblings in the California Chinook salmon population with the lowest heterozygosity (SRW) at a FNR of 0.05.

Half‐sibling (HS) pairs cannot be accurately distinguished from unrelated pairs in any populations without very large FNRs and in very modest‐sized studies with very few pairwise comparisons.

**FIGURE 4:**
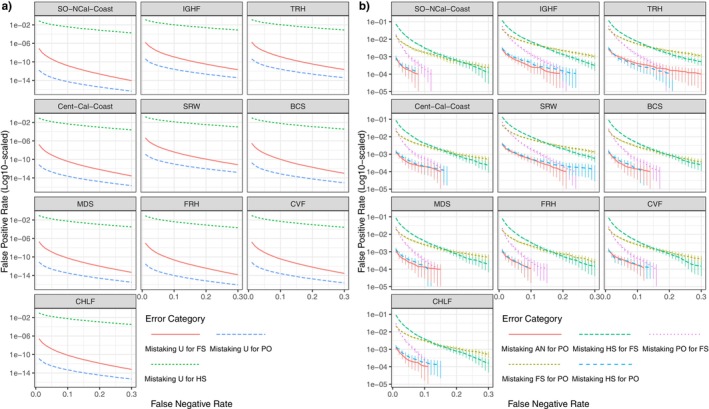
False‐positive rates (FPRs) as a function of false negative rates (FNRs) for distinguishing between different relationships. Groupings for panels that are neither a previously defined reporting unit nor a single collection (Table 2) are as follows: TRH = {TRS, TRF}; FRH = {FRHS, FRHF}; CVF = {BCF, MDF, SJRF}. (a) FPRs calculated using importance sampling of unrelated pairs of individuals being incorrectly categorized as parent‐offspring (PO), full‐sibling (FS) or half‐sibling (HS) pairs. (b) FPRs calculated using regular Monte Carlo sampling of avuncular pairs (AN: aunt‐niece, uncle‐nephew, etc.) or full‐siblings (FS) or half‐siblings (HS) being incorrectly categorized as parent‐offspring (PO); as well as FPRs of parent‐offspring (PO) pairs or half‐sibling (HS) pairs being incorrectly inferred as full siblings (FS). In both a and b, 100,000 Monte Carlo replicates were simulated.

In a reasonably large population, the vast majority of pairs of individuals are expected to be unrelated, or at least, effectively unrelated, sharing common ancestors only many generations in the past. However, a small fraction of pairs will be more recently related, and distinguishing these kin pairs from target relationships (like PO and FS) must be accounted for. “CKMRsim” provides facilities for assessing error rates between different kin groups, and results for these are summarized in (Figure 4b). Here, again, focusing on the results for the genetically depauperate SRW, and using an FNR of 0.1, the chance of incorrectly categorizing an HS as an FS, or an FS as a PO is less than 1 in 100. Likewise, we can expect PO to be misidentified as FS at a rate of around 1 in 500, and HS or Aunts/Uncles to be misidentified as parents at a rate less than 1 in 1000. Though these rates are much higher than the FPRs for unrelated pairs, they should be compared to the number of actual nontarget kin pairs expected. For example, in a salmon population, the number of full aunts or uncles of a fish is unlikely to exceed 50, at later life stages, so a FPR of < 0.001 is comfortably low in that case. Estimation of error rates between different kin‐categories cannot be done in “CKMRsim” using importance sampling. Consequently, it is difficult to accurately estimate the small probabilities (10^−4^ or less) in Figure 4b, as indicated by the error bars, which represent the estimate ±2*s* where *s* is the estimated standard error of the mean of the Monte Carlo sample. However, as noted above, because there are far fewer related pairs than unrelated pairs, it is not typically essential to have accurate estimates of very low FPRs for related pairs.

**FIGURE 5:**
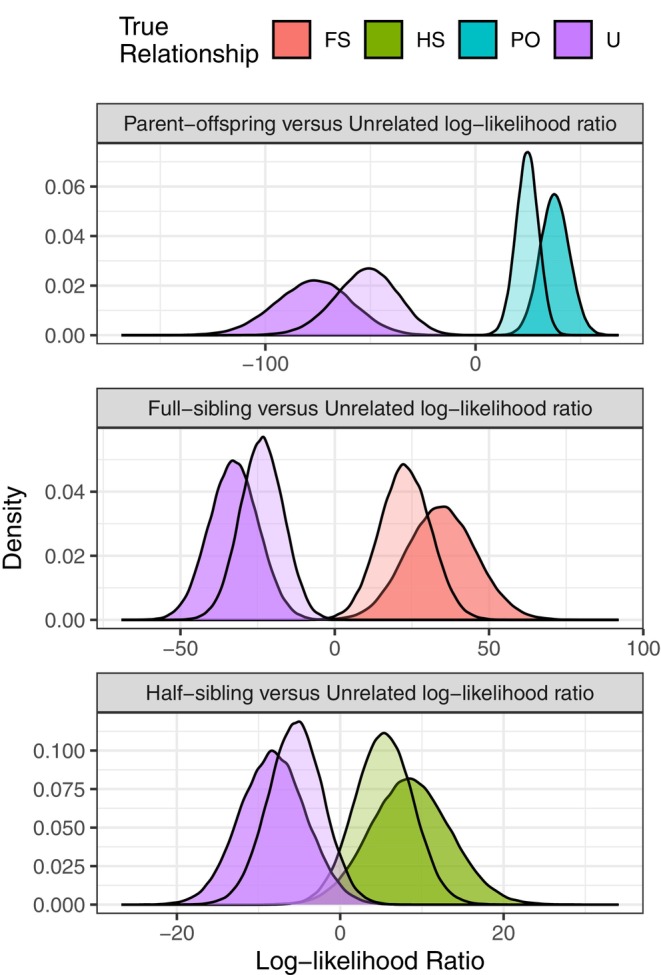
The distribution of log‐likelihood ratios for fish from Feather River Hatchery when scoring markers as microhaplotypes versus as single SNPs. Three different likelihood ratios are shown in the three panels. The opacity of the distributions indicates the marker type: The more opaque setting indicates markers scored as microhaplotypes; the more transparent setting indicates the use of only the most heterozygous SNP from among all SNPs in the microhaplotype. Evidently, scoring the markers as microhaplotypes produces more separation between the distributions for the relationships of PO, FS, or HS against the relationship of unrelated (U).

The presence of multiple alleles at many of these loci improves power for kin inference compared to a marker panel using only a single SNP at each amplicon. To provide a visual display of the difference afforded by using microhaplotypes compared to single SNPs in this dataset, we show the distribution of log‐likelihood ratios for different relationships using our microhaplotype‐scored amplicons versus using the most heterozygous single SNP from each amplicon for all fish from the FRH (Figure 5), with results for the PO versus Unrelated log‐likelihood ratio shown for more collections in Figure S8. The figures show a considerable increase in separation between the relationship categories from calling genotypes as microhaplotypes in these amplicons.

While there is not a currently available method (like that of Anderson and Garza 2006) for estimating error rates in the inference of parent‐offspring trios with multiallelic marker data, given the substantial amount of statistical power for identifying PO and FS pairs, which require much more statistical power, we hypothesize that PO‐trio inference with these markers will be very accurate.

## 4 Discussion

We describe a novel panel of microhaplotype genetic markers for Chinook salmon in California that provides sufficient power for highly accurate identification of parent and offspring pairs and full siblings, and also allows near‐perfect identification of individuals to population or genetic group of origin in California. This set of genetic markers includes multiallelic gene regions with high variability for relationship inference, gene regions identified in whole‐genome sequence data for increased power for identification of specific populations, and markers that have long been in use for both GSI and PBT in California (Clemento et al. 2014), by converting them into so‐called “SNPlicons,” which are treated as multiallelic haplotypes in the presence of additional variation surrounding the originally assayed SNP. Our work adds to a growing number of recent studies finding that microhaplotype markers can provide additional power over single SNPs for both GSI (McKinney et al. 2017, 2022; Hargrove et al. 2024) and relationship inference (Baetscher et al. 2018; Bootsma et al. 2020; Delomas et al. 2024). This set of markers lays the foundation for a comprehensive genetic monitoring and evaluation effort that facilitates multiple types of inference and is flexible and extensible.

This marker set and baseline reference dataset provide excellent power for identifying fish from all of the Chinook salmon ESUs in California, as well as individual populations within those ESUs (Figure 2). The California Central Valley (CCV) is one of the largest river basins on the west coast of North America and drains the Sierra Nevada and southern Cascade mountain ranges. It has the highest diversity of recognized Chinook salmon ecotypes in the species range, with four named ecotypes, two of which are protected under the US Endangered Species Act. It is also the source of household water for tens of millions of California residents and millions of acres of arguably the most productive and valuable agriculture area in North America. As such, accurate identification of the distinct ecotypes of CCV Chinook salmon is of utmost importance for monitoring and evaluation of individual ecotypes, and for designing and implementing effective conservation and management actions. This has been challenging, given the recent common ancestors of these ecotypes (Clemento et al. 2014), and ongoing migration between subbasins where different ecotypes predominate.

We describe the first set of genetic markers that produces easily replicable data and that identifies all of the ecotypes in the CCV, including the late‐fall‐run Chinook salmon ecotype. The late‐fall‐run occurs only in the CCV and shares a genomic background with the more common CCV fall‐run salmon ecotype, so it has been refractory to previous GSI efforts with other marker types (Seeb et al. 2007; Clemento et al. 2014; Meek et al. 2016; Thompson et al. 2024).

Moreover, we show how the FRH spring‐run lineage is easily identifiable through a combination of traditional GSI and the characterization of functional genetic markers in the RoSA. Finally, although previous work has described GSI capabilities that distinguish the natural‐spawning CCV spring‐run lineages from each other and their fall‐run counterparts with moderately high accuracy, we demonstrate near complete accuracy in distinguishing these “stocks.” Moreover, the few fish that are apparently misidentified (Figure 2, Figure S6) are likely to be primarily migrants and not true misidentifications. For example, the three fish that were field characterized as spring‐run from Butte Creek, but are genetically identified as winter‐run salmon, clearly carry the winter‐run genomic background and are strays from the winter‐run stock, as these could not realistically be misidentified on the basis of the genetic data.

Results from the model‐based clustering analysis with the program STRUCTURE, revealed patterns of population relationships that are coincident with previous work (Clemento et al. 2014; Kinziger et al. 2013), emphasizing the distinction between Chinook salmon populations in Coastal California and the Central Valley. These analyses also uncover some additional patterns, including the clear presence of mixed ancestry in the Coastal California population in the Russian River, which is consistent with its location, at the southernmost edge of the Coastal Chinook salmon distribution and proximate to the mouth of the Sacramento River (the Golden Gate). In the Central Valley, the clustering results emphasize the genetic distinction of the SWR population, which is likely at least partially due to the extreme bottleneck that it passed through in the 1980s and 1990s (Hedrick and Hedgecock 1994). The inclusion of the LFAR markers also resulted in the clear distinction of the CHLF group, which is coincident with the long‐known phenotypic distinction of this population, but represents a novel genetic result. Moreover, at *K* = 7, the fall‐run reporting group breaks into two clusters, which are distributed across almost all of the spring and fall‐run populations in the Central Valley, albeit not equivalently, emphasizing both current and historical migration and gene flow between them.

Examination of the geographic patterns of allele frequencies for the RoSA‐associated loci found a clear instance of the early‐migrating haplotype present in the California Coastal Chinook salmon lineage. This is of note because this lineage does not currently have an early‐migrating component, although it has long been speculated that the Eel River, at least, historically harbored early‐migrating Chinook salmon, and early‐migrating steelhead ( *Oncorhynchus mykiss* ) persist in the basin (Bjorkstedt et al. 2005). Moreover, we discovered several recombinant RoSA haplotypes in both of the CCC populations that indicate a long‐standing presence of both the early‐ and late‐migrating haplotypes in these populations. This is consistent with the idea that early‐migrating populations arise through migration of small numbers of individuals carrying E‐lineage haplotypes, in either heterozygous or homozygous form, which are subsequently positively selected when appropriate habitat conditions exist. A similar pattern of recombination, due to long‐standing co‐occurrence of the E and L haplotypes in heterozygotes, has been previously documented in the Klamath River (Thompson et al. 2020).

Previous genetic marker sets for California Chinook salmon had sufficient power for inferring parent‐offspring relationships, but only when both parents were sampled and genotyped (Clemento 2013). This marker set considerably increases the capacity for relationship inference in California salmon by providing sufficient power for parent‐offspring relationship inference when only one parent is sampled and identifying pairs of full siblings when no parents are sampled. Groups of full siblings larger than two can be identified even more accurately than expressed in Figure 5 when using statistical approaches that account for the joint relationships between more than two individuals, such as COLONY (Wang 2004). Although some additional errors might be expected when related but nontarget kin pairs are sampled, the FPRs for these nontarget kin pairs are sufficiently small for almost all realistic scenarios. Accurate relationship inference in even the most genetically depauperate Chinook salmon population in California and rangewide (Seeb et al. 2007; Clemento et al. 2014) is therefore possible with this marker set. We note that, as in previous work, it is nearly impossible to identify HS pairs accurately with data from this or any standard marker dataset. Accurate identification of HS pairs typically relies on many hundreds of microhaplotype markers (Baetscher et al. 2018) or thousands of SNP markers (Hillary et al. 2018).

As sequencing costs drop, whole‐genome sequence (WGS) data may become the preferred data type for salmon genetics. If so, it may become possible to include all the > 200 genetically distinct Chinook salmon populations in North America within a single standardized reference baseline constructed with WGS data that performs equally well at broad and regional scales (DeSaix et al. 2024). Such an approach would be highly flexible and extensible, as it would allow for the assignment of unknown‐origin fish using just about any marker type, including reduced‐representation DNA sequencing (e.g., RADseq, Meek et al. 2020; Thompson et al. 2024), as the variation used by such region‐specific panels of markers should be contained within the WGS baseline dataset. For the present, however, baselines tailored to specific regions are essential for regional management questions, and targeted sequencing approaches have proven to be the most practical for large‐scale applications of either GSI, PBT, or an integrated monitoring program (Beacham et al. 2021).

## Conflicts of Interest

The authors declare no conflicts of interest.

## Supporting information


Appendix S1

Data S1

## Data Availability

All data and code needed to reproduce the results here are available online. Online version of data and scripts used in paper: https://github.com/eriqande/california‐chinook‐microhaps. Archived version of data and scripts used in paper: https://doi.org/10.5281/zenodo.15271275. Data repository with full reference dataset: https://doi.org/10.5061/dryad.hqbzkh1v2.
