# Supplementary material for: A Multipurpose Microhaplotype Panel for Genetic Analysis of California Chinook Salmon
Source: Evol Appl. 2025 May 13;18(5):e70110. doi: 10.1111/eva.70110 (PMC12070256; doi:10.1111/eva.70110)
Supplement: Supplementary file 1 — Appendix S1 [file EVA-18-e70110-s001.pdf]

SUPPLEMENT TO: A multipurpose microhaplotype panel for genetic analysis of California Chinook salmon

Eric C. Anderson[1,§], Anthony J. Clemento[1,2], Matthew A. Campbell[1,2†], Devon E. Pearse[1,2], Anne K. Beulke[1,3], Cassie Columbus[1,2], Ellen Campbell[1,2], Neil F. Thompson[1,2‡], John Carlos Garza[1,2,§]

[1]*Southwest Fisheries Science Center, National Marine Fisheries Service, NOAA, Santa Cruz, California, USA.* [2]*Institute for Marine Sciences, University of California, Santa Cruz, USA.* [3]*Department of Ocean Sciences, University of California, California, Santa Cruz, USA.* [†]*Current address: Centre for Carbon, Water and Food, The University of Sydney, 380 Werombi Road, NSW 2570, Australia.* [‡]*Current address: Pacific Shellfish Research Unit, Agricultural Research Service, US Department of Agriculture, Newport, Oregon, USA.*

[§]Correspondence: eric.anderson@noaa.gov, carlos.garza@noaa.gov

Contents

S1 Winter-run-associated polymorphism methods and results 2

List of Figures

S1 Genomic locations of amplicons . . . 3
S2 Negative log base 10 of association p-values for individual SNPs for late-fall versus fall run 4
S3 Candidates chosen for assay design for the late-fall associated region . . . 4
S4 Number of loci with different total numbers of alleles . . . 5
S5 STRUCTURE minor modes found by CLUMPAK . . . 5
S6 Assignment table for fish with scaled likelihood > 0.8 . . . 6
S7 Assignment table by RoSA genotype . . . 7
S8 Comparison of microhaplotypes vs. best SNP in each amplicon . . . 8
S9 Absolute difference in allele frequency between winter run and non-winter run fish . . . 9
S10 Sliding windows of absolute allele frequency difference between winter run and others . . 10
S11 61 candidates for primer design for winter-run-associated polymorphisms . . . 11

List of Tables

S1 Microhaplotype discovery panel sample sizes . . . 12
S2 Allele frequencies of winter-run-associated polymorphisms . . . 12

S1 Winter-run-associated polymorphism methods and results

We used the whole genome sequencing data from Thompson *et al.* (2020) to seek variants with large allele frequency differences between winter-run Chinook salmon and all the other Chinook salmon ecotypes in the Central Valley of California (CCV). Because winter-run Chinook salmon are already highly differentiated from all others, we did not pursue an association study as used for identifying the late-fall-run associated variants. Rather, we first identified regions with a high density of variants with large allele frequency differences between winter-run and non-winter-run fish in the CCV. Subsequently we identified SNPs within those regions with particularly large allele frequency differences. This approach was taken to avoid targeting single, isolated SNPs with large allele frequency differences that may have resulted merely from sampling variation, which was a concern because we had whole genome sequencing data from only 16 winter run fish.

More specifically, we calculated allele frequencies for 16 winter-run fish and 84 non-winter-run fish from the Thompson *et al.* (2020) variant data VCF files using ANGSD version 0.921. Sites were retained if at least 62.5% of samples in each group had read data (10 of 16 for the winter run and 50 of 80 for the non-winter run), resulting in 7,295,001 SNPs for downstream analysis. We first investigated the distribution throughout the genome of $|d|$, the absolute difference of the alternate allele frequency between the two groups (Figure S9). This revealed that many loci had large values of $|d|$, but there were several regions in the genome, in particular, with prominent peaks in allele frequency difference and one or more SNPs apparently fixed for alternate alleles between the two sample groups.

To leverage information from multiple SNPs to identify regions in the genome with large allele frequency differences, we calculated the fraction of SNPs with $|d| > 0.5$ that also had $|d| > 0.9$ within non-overlapping 100 kb sliding windows throughout the genome. This metric indicated several prominent peaks. We focused on all of those 100 kb windows in which more than 12.4% of sites with $|d| > 0.5$ also had $|d| > 0.9$ which were also adjacent to at least one window in which $> 10\%$ of sites with $|d| > 0.5$ also had $|d| > 0.9$ (Figure S10). Within the windows found on four different chromosomes using the above criteria, we then attempted to design amplicons to type the SNPs at a subset of the sites within each window. We chose all sites with $|d| > 0.975$, as well as the 8 SNPs on each chromosome with the highest values of $|d|$, yielding 61 candidate SNPs (Figure S11).

Some of those 61 candidate SNPs were close enough that it was possible to consider amplifying them with PCR on 58 different short sequences. We used Primer3 (Untergasser *et al.*, 2012) to return three possible primer-pair designs for each of the 58 sequences and then chose the primer pair with the fewest penalties, optimal target size, and most consistent melting temperatures. One primer pair was dropped from consideration because it amplified a large indel that was apparent in the sequence data which would have rendered the sequence too large to efficiently amplify and several others were dropped because the primers overlapped other amplicons. Finally, several amplicons with the lowest $|d|$ on chromosome 16 (RefSeq NC_037112.1) were removed from consideration, leaving us with 48 amplicons to test for amplification and for evaluation of allele frequencies.

These 48 amplicons were amplified and sequenced in 192 fish—96 Feather River spring run and 96 winter run—on a MiSeq sequencer, and the variants were called using GATK. The resulting VCF file was used to estimate allele frequency differences between winter run and Feather River spring run. We also processed the sequence data using the R package, 'microhaplot' (`https://github.com/ngthomas/microhaplot`), and visually inspected loci for consistent allele depth ratio and numbers of haplotypes. We then chose 24 amplicons for further testing on the basis of allele frequency differences between Feather River spring run and winter run, number of haplotypes, and ease of scoring. These 24 markers were typed on a variety of fish over the course of a year, and we finally chose three to include in our California Chinook reference baseline: one amplicon on each of chromosomes 8, 12, and 16. The estimated frequencies of all the alleles present in the reference baseline in those three amplicons shows that there are not fixed differences at these markers between winter run and all other reporting units (Table S2).

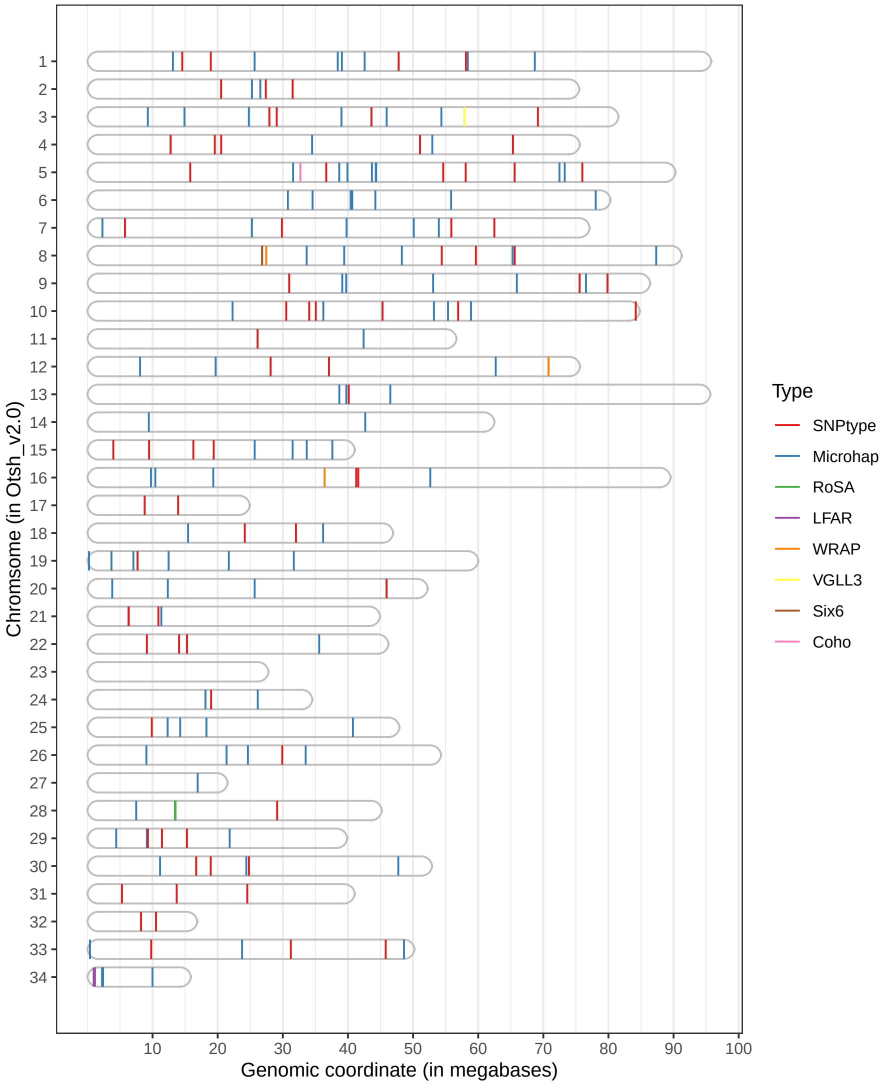

Figure S1 Genomic locations of amplicons in the Otsh_v2.0 assembly of the Chinook salmon genome. Color shows type of marker (see main text). The sex-ID marker is not included here because it aligns to a scaffold that is not part of a named chromosome/linkage group in the assembly.

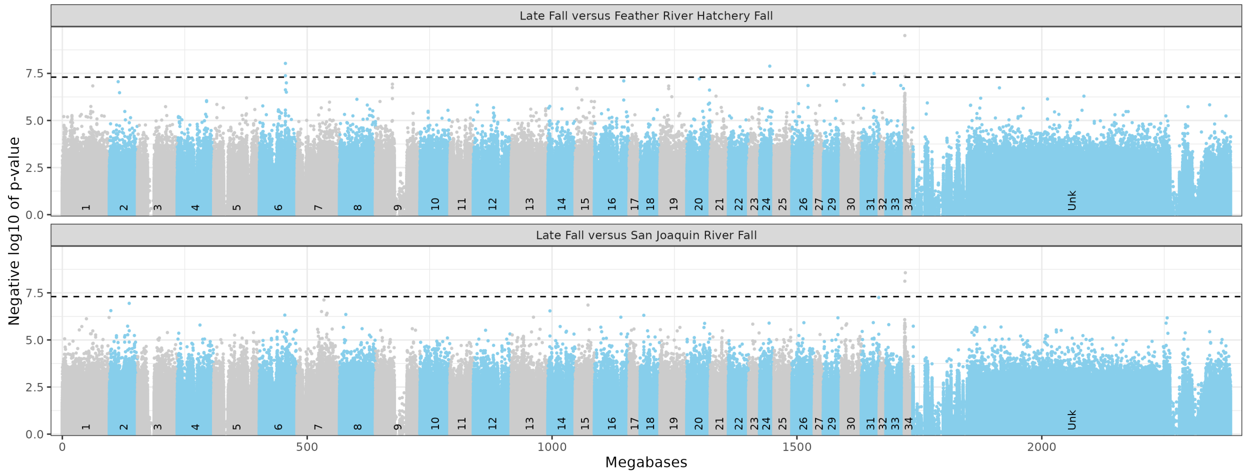

Figure S2 Negative log base 10 of association p-values for individual SNPs for late-fall versus fall run. x axis shows position in genome (in megabases), with color alternating by chromosome, as indicated by numbers above the x-axis. "Unk" refers to unplaced scaffolds in the Otsh_v1.0 genome assembly (Christensen *et al.*, 2018). The upper panel is the comparison between late-fall and Feather River Hatchery fall, while the lower panel is the comparison of late-fall to San Joaquin River fall. The dashed line corresponds to a significance threshold of 5×10^{-8}. The only SNPs exceeding that threshold in both comparisons are the ones atop the prominent peaks on chromosome 34.

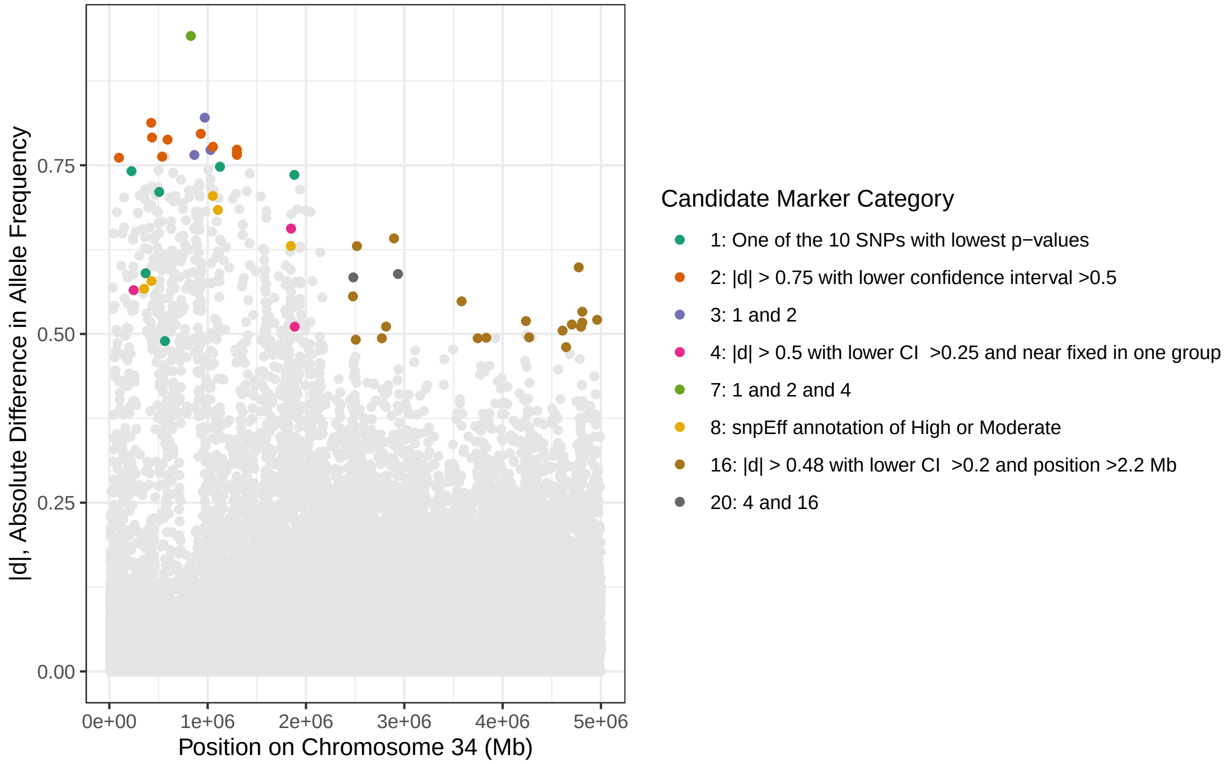

Figure S3 Positions (on x axis) and absolute allele frequency difference between whole-genome sequenced fall-run and late-fall run fish (on y axis) of 49 candidate SNPs for which amplicons were designed. Gray points are SNPs that were not candidates. Colors of points denote the reason each SNP was chosen as a candidate. See main text "Methods" for further explanation of categories.

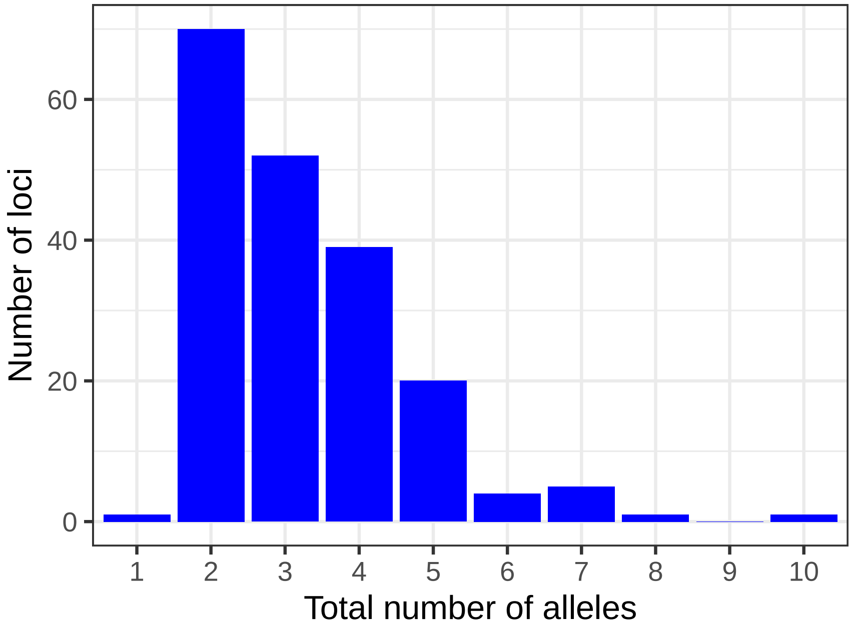

Figure S4 Number of loci with different total numbers of alleles in the data set. The one amplicon with only one allele is `Ots_coho001_05_32691399`, which is fixed for alternate alleles in Chinook vs. coho salmon. It is helpful in identifying coho samples that are misidentified during sampling as Chinook salmon.

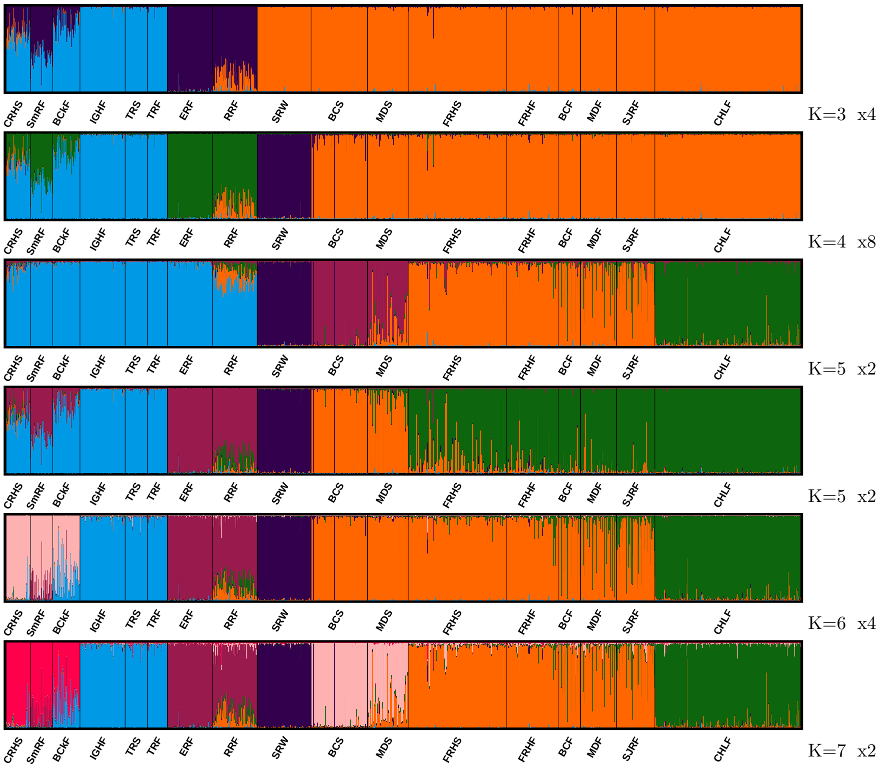

Figure S5 STRUCTURE minor modes found by CLUMPAK. At each value of K for which a minor mode was found, the plot is shown. K values and number of times each minor mode appeared out of 20 replicate runs of STRUCTURE appear to the lower right of each.

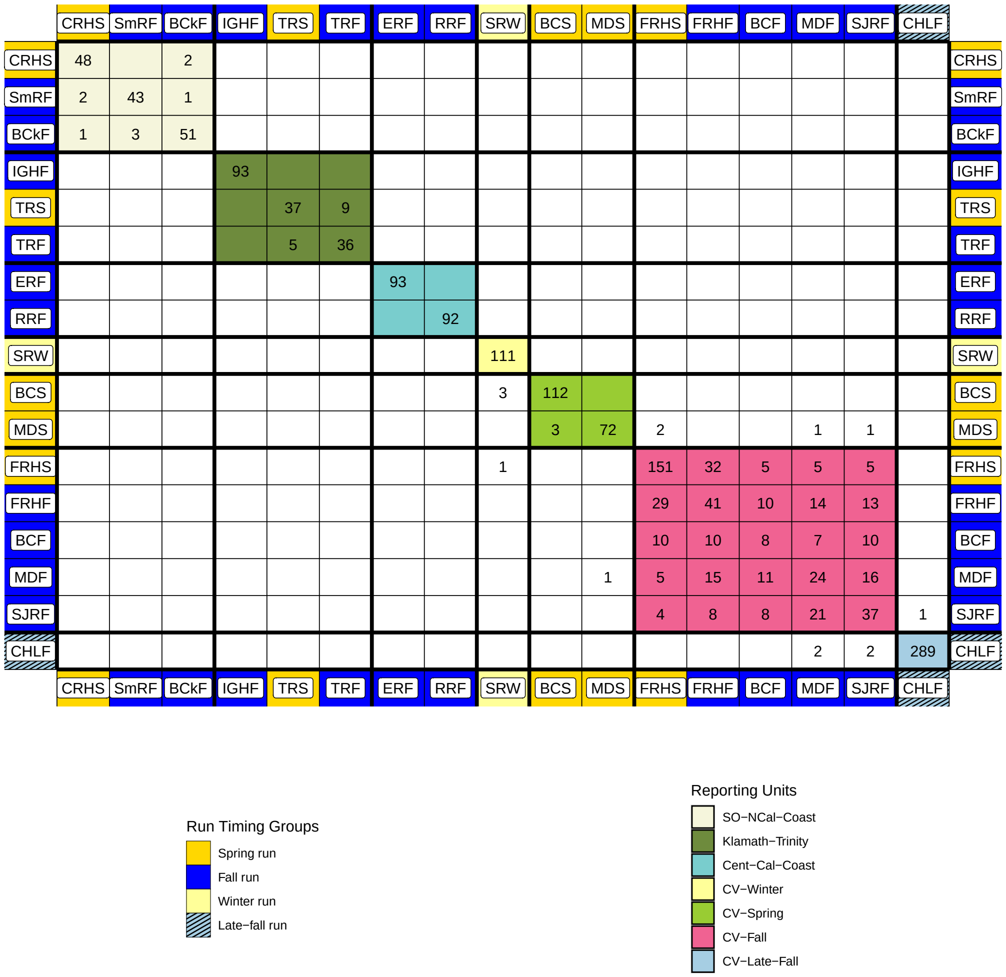

	CRHS	SmRF	BCkF	IGHF	TRS	TRF	ERF	RRF	SRW	BCS	MDS	FRHS	FRHF	BCF	MDF	SJRF	CHLF	
CRHS	48		2															CRHS
SmRF	2	43	1															SmRF
BCkF	1	3	51															BCkF
IGHF				93														IGHF
TRS					37	9												TRS
TRF					5	36												TRF
ERF							93											ERF
RRF								92										RRF
SRW									111									SRW
BCS									3	112								BCS
MDS										3	72	2			1	1		MDS
FRHS									1			151	32	5	5	5		FRHS
FRHF												29	41	10	14	13		FRHF
BCF												10	10	8	7	10		BCF
MDF											1	5	15	11	24	16		MDF
SJRF												4	8	8	21	37	1	SJRF
CHLF															2	2	289	CHLF
	CRHS	SmRF	BCkF	IGHF	TRS	TRF	ERF	RRF	SRW	BCS	MDS	FRHS	FRHF	BCF	MDF	SJRF	CHLF	

Figure S6 Assignment table like that in Figure 2b in the paper, but constrained so that only fish assigning to a reporting unit with scaled likelihood greater than 0.8 are included.

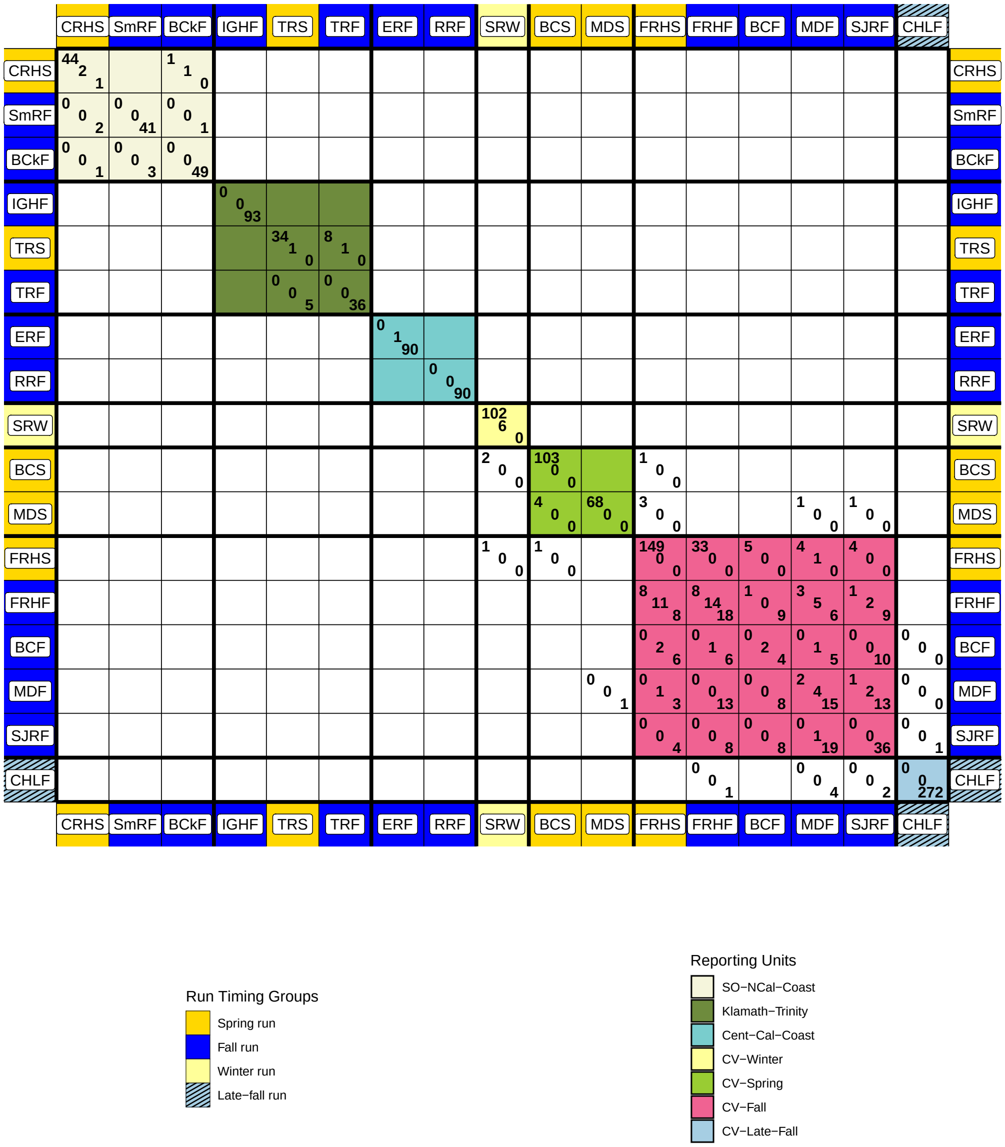

Figure S7 Assignment table like that in Figure 2b in the paper, but with numbers according to genotypes at the RoSA. In each cell, the top left entry gives the number of EE (early run allele homozygotes) genotypes, the middle entry is the number of EL genotypes (heterozygotes), and the bottom right entry gives the number of LL (late-run homozygote) genotypes.

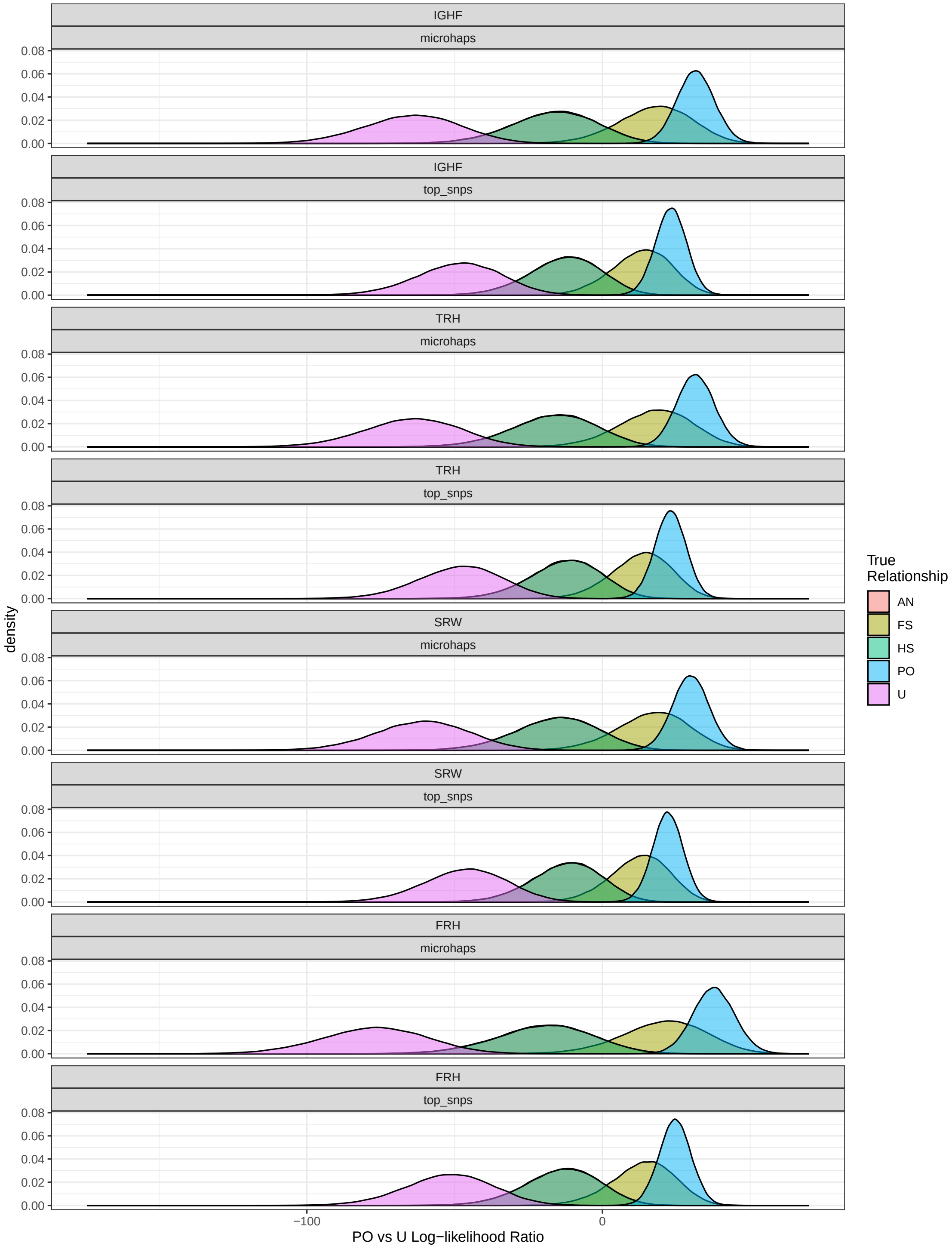

Figure S8 A comparison of the distribution of log-likelihood ratios when using all the alleles at each amplicon typed as microhaplotypes ("microhaps" in the panel headers) versus using just a single (the most informative) SNP from each amplicon ("top_snps" in the panel headers). Results shown for four hatchery collections in California (FRH: Feather River Hatchery [spring and fall]; IGH: Iron Gate Hatchery; SRW: Sacramento Winter Run; TRH: Trinity River Hatchery [spring and fall]). The density plots show the distribution of log-likelihood ratios for Parent-Offspring vs Unrelated for four different relationships: PO = parent offspring; FS = full-sibling; HS = Half sibling; AN = avuncular (i.e., aunt-niece, etc.) The distributions for AN and HS overlap completely. Note that the overlap between FS and PO occurs for the PO vs U likelihood ratio, but is nearly eliminated with the PO vs FS likelihood ratio, allowing these two relationships to be resolved accurately.

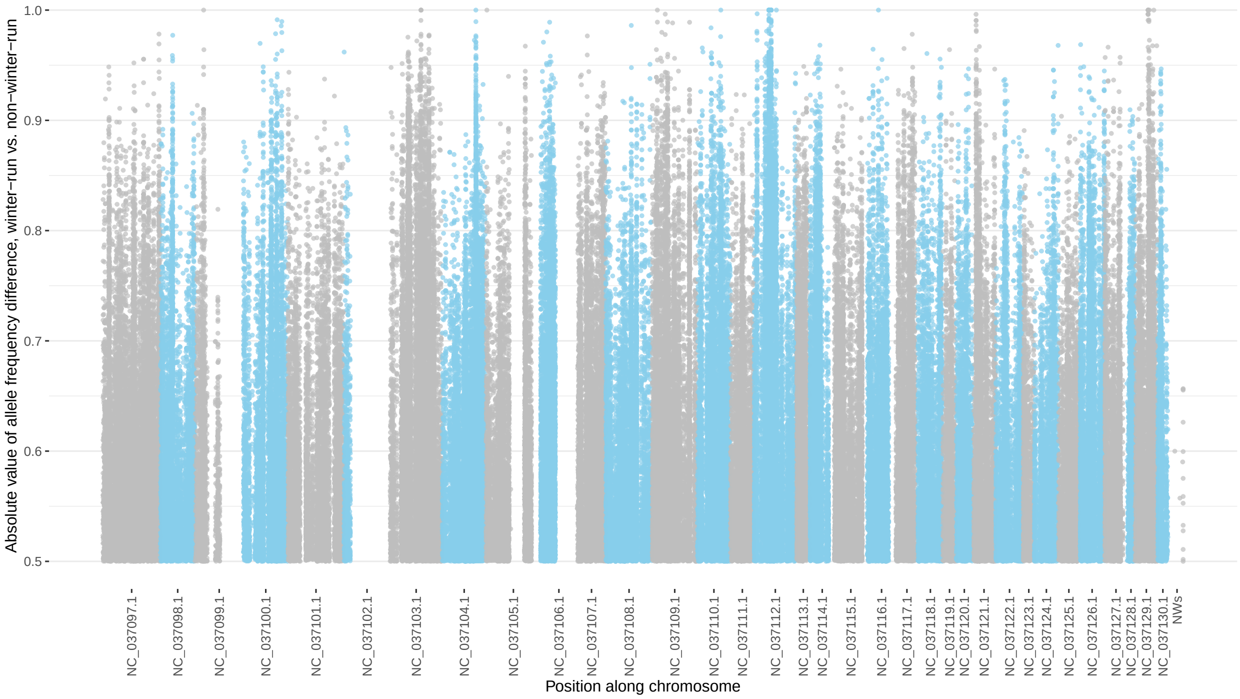

Figure S9 Absolute difference in allele frequency between winter run and non-winter run fish of the CCV. The plot shows only those SNPs with at least a frequency difference of 0.5 between the two groups. Each point is a SNP. The x axis shows position in the Otsh_v1.0 genome with color alternating by chromosome as indicated by RefSeq names on plot.

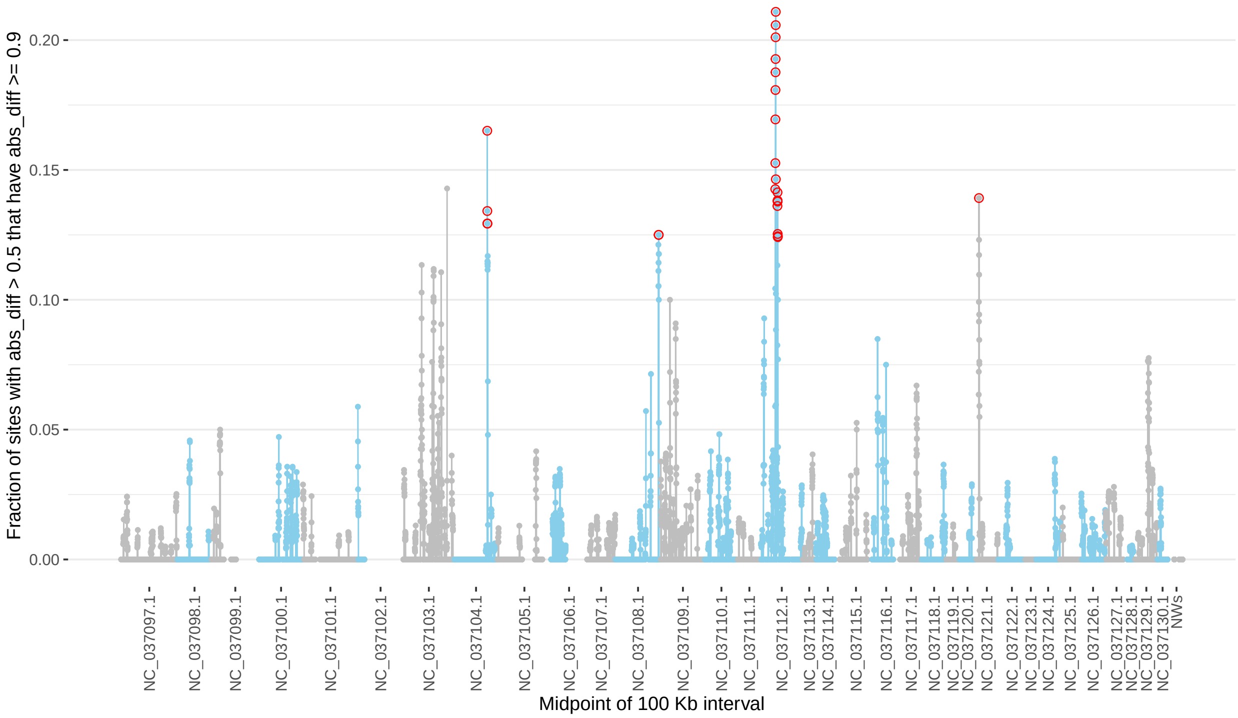

Figure S10 Values within 100 kb sliding windows of the fraction of sites with $|d|$ (absolute difference in allele frequency between winter run and non-winter run fish of the CCV) greater than 0.5 that also have $|d| > 0.9$. The x axis shows position in the Otsh_v1.0 genome with color alternating by chromosome as indicated by RefSeq names on plot. Red circles denote sliding windows chosen for further investigation for candidate markers.

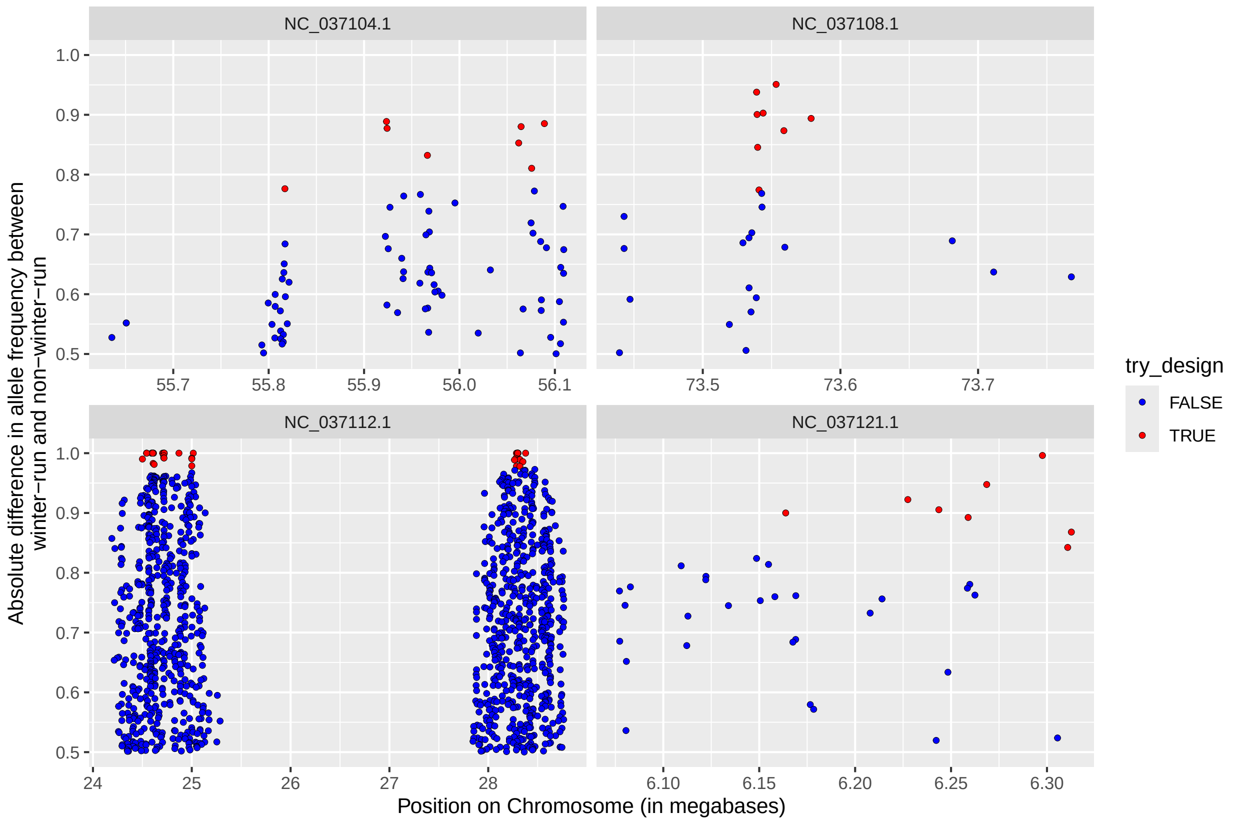

Figure S11 Genomic positions and values of $|d|$ (absolute difference in allele frequency between winter run and non-winter run fish of the CCV) for the 61 candidate SNPs (in red) to design for winter-run-associated polymorphisms (WRAPs). Other sites are shown in blue. The x-axis shows position on each chromsome in the Otsh_v1.0 assembly. The chromsome name is indicated in the facet headers by RefSeq name

Table S1 Numbers of fish from different collections/populations used in the microhaplotype discovery effort. N is the number of diploid individuals included in the ascertainment panel.

Population	N
Sacramento River winter run	4
Feather River spring run	2
Feather River fall run	2
Butte Creek spring run	2
Deer Creek fall run	2
Eel River fall run	2
Klamath Basin, Trinity River Hatchery spring run	2
Southern Oregon, Chetco River	2
Northern Oregon, Siletz River	2
Columbia River, Kalama Hatchery spring run	2
Upper Columbia River, Wenatchee River	2
Snake River, McCall Hatchery summer-spring run	2
Puget Sound,Kendall Hatchery spring run	2
Fraser River, Thompson River, Spius Creek	2
Fraser River, Birkenhead River	2

Table S2 Allele frequencies across reporting units of the three winter-run associated markers. Genome coordinates of SNPs are from Otsh_v2.0. Alleles are listed according to the SNP nucleotide at the variable SNPs within the amplicon.

Locus	Allele	CV-Winter	CV-Spring	CV-Fall	CV-Late-Fall	Cent-Cal-Coast	Klamath-Trinity	SO-NCal-Coast
Chr08:27450181	C	0.838	0.257	0.088	0.039	0.015	0.022	0.016
Chr08:27450181	A	0.162	0.743	0.912	0.961	0.985	0.978	0.984
Chr12:70794116	GAA	0.815	0.032	0.033	0.017	0.000	0.000	0.000
Chr12:70794116	GAT	0.149	0.860	0.817	0.734	0.700	0.720	0.686
Chr12:70794116	AAT	0.032	0.087	0.142	0.234	0.022	0.026	0.024
Chr12:70794116	GTT	0.005	0.021	0.008	0.015	0.278	0.254	0.291
Chr16:36409831	CCG	0.797	0.026	0.007	0.003	0.000	0.009	0.192
Chr16:36409831	TCG	0.162	0.263	0.336	0.437	0.195	0.430	0.371
Chr16:36409831	TCA	0.036	0.616	0.610	0.507	0.485	0.547	0.415
Chr16:36409831	TGG	0.005	0.095	0.048	0.053	0.320	0.015	0.022

References

Christensen KA, Leong JS, Sakhrani D, Biagi CA, Minkley DR, Withler RE, Rondeau EB, Koop BF, Devlin RH (2018) Chinook salmon (Oncorhynchus tshawytscha) genome and transcriptome. *PloS one*, **13**, e0195461.

Thompson NF, Anderson EC, Clemento AJ, Campbell MA, Pearse DE, Hearsey JW, Kinziger AP, Garza JC (2020) A complex phenotype in salmon controlled by a simple change in migratory timing. *Science*, **370**, 609–613.

Untergasser A, Cutcutache I, Koressaar T, Ye J, Faircloth BC, Remm M, Rozen SG (2012) Primer3—new capabilities and interfaces. *Nucleic Acids Research*, **40**, e115–e115.
